#  Alternative splicing of *Arabidopsis G6PD5* recruits NADPH-producing OPPP reactions to the endoplasmic reticulum

**DOI:** 10.3389/fpls.2022.909624

**Published:** 2022-09-02

**Authors:** Loreen Linnenbrügger, Lennart Doering, Hannes Lansing, Kerstin Fischer, Jürgen Eirich, Iris Finkemeier, Antje von Schaewen

**Affiliations:** ^1^Department of Biology, Molecular Physiology of Plants, Institute of Plant Biology and Biotechnology, University of Münster (WWU Münster), Münster, Germany; ^2^Department of Biology, Plant Physiology, Institute of Plant Biology and Biotechnology, University of Münster (WWU Münster), Münster, Germany

**Keywords:** alternative splicing, NADPH formation, endoplasmic reticulum, cytosolic G6PD isoforms, Arabidopsis, metabolons, oxidative pentose phosphate pathway

## Abstract

Glucose-6-phosphate dehydrogenase is the rate-limiting enzyme of the oxidative pentose-phosphate pathway (OPPP). The OPPP mainly provides NADPH and sugar-phosphate building blocks for anabolic pathways and is present in all eukaryotes. In plant cells, the irreversible part of the OPPP is found in several compartments. Among the isoforms catalyzing the first OPPP step in Arabidopsis, G6PD1 to G6PD4 target plastids (with G6PD1 being also directed to peroxisomes), whereas G6PD5 and G6PD6 operate in the cytosol. We noticed that alternative splice forms *G6PD5.4* and *G6PD5.5* encode N-terminally extended proteoforms. Compared to *G6PD5.1*, RT-PCR signals differed and fluorescent reporter fusions expressed in Arabidopsis protoplasts accumulated in distinct intracellular sites. Co-expression with organelle-specific markers revealed that the G6PD5.4 and G6PD5.5 proteoforms label different subdomains of the endoplasmic reticulum (ER), and analysis of C-terminal *ro*GFP fusions showed that their catalytic domains face the cytosol. In *g6pd5-1 g6pd6-2* mutant protoplasts lacking cytosolic G6PDH activity, the ER-bound proteoforms were both active and thus able to form homomers. Among the Arabidopsis 6-phosphogluconolactonases (catalyzing the second OPPP step), we noticed that isoform PGL2 carries a C-terminal CaaX motif that may be prenylated for membrane attachment. Reporter-PGL2 fusions co-localized with G6PD5.4 in ER subdomains, which was abolished by Cys-to-Ser exchange in the ^256^CSIL motif. Among the Arabidopsis 6-phosphogluconate dehydrogenases (catalyzing the third OPPP step), S-acylated peptides were detected for all three isoforms in a recent palmitoylome, with dual cytosolic/peroxisomal PGD2 displaying three sites. Co-expression of GFP-PGD2 diminished crowding of OFP-G6PD5.4 at the ER, independent of PGL2's presence. Upon pull-down of GFP-G6PD5.4, not only unlabeled PGD2 and PGL2 were enriched, but also enzymes that depend on NADPH provision at the ER, indicative of physical interaction with the OPPP enzymes. When membrane-bound G6PD5.5 and 5.4 variants were co-expressed with KCR1 (ketoacyl-CoA reductase, involved in fatty acid elongation), ATR1 (NADPH:cytochrome-P450 oxidoreductase), or pulled C4H/CYP73A5 (cinnamate 4-hydroxylase) as indirectly (*via* ATR) NADPH-dependent cytochrome P450 enzyme, co-localization in ER subdomains was observed. Thus, alternative splicing of *G6PD5* can direct the NADPH-producing OPPP reactions to the cytosolic face of the ER, where they may operate as membrane-bound metabolon to support several important biosynthetic pathways of plant cells.

## Introduction

In the past decade, the study of metabolic pathways has seen a revival, specifically the one of NADPH formation by the oxidative pentose-phosphate pathway (OPPP; Stincone et al., 2015). NADPH is a reduction equivalent needed for anabolic biosynthesis and redox homeostasis in different cellular compartments of all eukaryotes. Hence, the main focus of our studies is the localization of the irreversible OPPP reactions in plant cells, which starts with branching from glycolysis at the level of glucose-6-phosphate by glucose-6-phosphate dehydrogenase (G6PDH). G6PDH is active as a dimer or tetramer and catalyzes the first OPPP step followed by 6-phosphogluconolactonase (6PGL, which is active as monomer), and 6-phosphogluconate dehydrogenase (6PGDH, active as dimer). Together, they catalyze the formation of ribulose-5-phosphate (Ru5P, important precursor of nucleotides) and two moles of NADPH (by the two dehydrogenases) at the expense of CO_2_ (Kruger and von Schaewen, 2003).

Due to partial overlap with the reductive pentose-phosphate pathway, better known as Calvin–Benson–Bassham (CBB) cycle in photoautotrophic organisms and tissues, the irreversible OPPP part in the cytosol is linked to the full cycle in plastids (Schnarrenberger et al., 1995). The exchange of intermediates occurs *via* the outer and inner envelope membranes, i.e., by porins (Duy et al., 2007) and sugar-phosphate/phosphate antiporters, respectively (Weber et al., 2005). In the case of OPPP metabolites, transport is mediated by outer envelope protein OEP21 and in the inner envelope by xylulose-5-phosphate transporter XPT (after epimerization of Ru5P to xylulose-5-phosphate), or glucose-6-phosphate transporters GPT1 and GPT2, with GPT1 also exchanging Ru5P for G6P (Baune et al., 2020).

In chloroplasts, the OPPP mainly operates in the dark. In the light, plastidial G6PDH enzymes are inactivated by disulfide–dithiol interchange of a redox-sensitive disulfide bridge (Scheibe, 1990; Wenderoth et al., 1997; Wendt et al., 1999, 2000). Like the pace-making enzymes of the CBB, plastidial G6PDH is a target of the ferredoxin–thioredoxin (Fd/Trx) system, but inversely regulated to prevent futile cycles. Conversely, CBB key enzymes such as phosphoribulokinase (PRK), glyceraldehyde 3-phosphate dehydrogenase (GAPDH), fructose-1,6-bisphosphatase (FBPase), and sedoheptulose-1,7-bisphosphatase (SBPase) are active in the light, but inactive in the dark. The Calvin cycle enzymes are organized as multi-enzyme complex (reviewed by Winkel, 2004) with the small CP12 protein serving as a platform for PRK and GAPDH sequestration (Wedel and Soll, 1998; Graciet et al., 2003). Metabolic channeling has also been proposed for the two OPPP dehydrogenases in plastids (Debnam et al., 1997); yet during high flux, an active lactonase seems also to be needed in both plastids and peroxisomes (i.e., plastidial/peroxisomal PGL3 and not cytosolic/peroxisomal PGL5; Lansing et al., 2020).

The two cysteine residues that are responsible for redox regulation of G6PDH activity in plastids were elucidated with recombinant enzymes from potato (P1 class, Wenderoth et al., 1997; P2 class, Wendt et al., 2000). This mechanism was later confirmed for the Arabidopsis G6PD isoforms (Wakao and Benning, 2005), with details for redox regulation by different plastidial thioredoxins (Née et al., 2009). The redox switch of Trx *m2* seems to play an additional role in alternative targeting of G6PD1 to peroxisomes, leading to cytosolic retention, which involves the catalytically inactive but evolutionary conserved G6PD4 isoform (Meyer et al., 2011). Actually, cytosolic retention extends to dual plastidial/peroxisomal PGL3 that catalyzes the 2nd OPPP step: Trx *m2* (retained by an as yet unknown mechanism in the cytosol) binds as co-chaperon to plastidial OPPP precursors in the cytosol. Upon Trx redox switch from *holdase* to *foldase* function, alternative targeting of PGL3 (and G6PD1) to peroxisomes is achieved (Hölscher et al., 2014). Importantly, only folded proteins are imported by peroxisomes (reviewed in Hu et al., 2012). Dual targeting was also found for the Arabidopsis PGD isoforms (catalyzing the 3rd OPPP step), with PGD1 and PGD3 found to reside in the cytosol and plastids, and PGD2 in the cytosol and peroxisomes (Hölscher et al., 2016). Finally, the GPT1 transporter does not only target plastids, but also the endoplasmic reticulum (ER), from where the protein can be recruited to peroxisomes to mediate G6P-Ru5P exchange (Baune et al., 2020).

Stress or developmental change activates glycolysis and the OPPP, which provides NADPH for oxidative bursts at the plasma membrane (Pugin et al., 1997; Scharte et al., 2009). Upon post-translational activation (phosphorylation) of reactive burst oxidase homologs (Rboh), superoxide (·O${}_{2}^{-}$) is extruded into the acidic apoplast of plant cells, where it is converted to hydrogen peroxide (H_2_O_2_), and may enter the cytosol *via* aquaporins to initiate redox signaling (Mittler, 2017). Of note, the glutathione pool mainly recovers *via* NADPH-dependent glutathione reductase and thus also profits from OPPP activation under stress. Moreover, besides catalase – acting as main H_2_O_2_ sink in peroxisomes (Van Breusegem et al., 2001) – H_2_O_2_ can be dissipated by peroxiredoxins (Prx). They retrieve electrons from Trx or glutaredoxins (Grx), thus activating cognate target enzymes. These chain reactions form the basis of redox signaling in most plant cell compartments and show extensive crosstalk with phosphorylation cascades in the cytosol (Dietz, 2014). In fact, phosphorylation of a conserved threonine residue stimulates the activity of Arabidopsis cytosolic G6PD6 and G6PD5 (Dal Santo et al., 2012), whereas slower responses involve sugar-sensing followed by transcriptional upregulation of the cytosolic *G6PD* isoforms (Hauschild and von Schaewen, 2003). In source leaves, sugars are retained upon stress – mainly due to the triggered formation of callose plugs at plasmodesmata (Scharte et al., 2009).

On top of these mechanisms, alternative splicing (AS) may additionally contribute to the described scenarios, since it can dramatically increase protein variation by either skipping exons or retaining introns (Reddy, 2007). In Arabidopsis, around 61% of the multi-exon genes are alternatively spliced under normal growth conditions (Marquez et al., 2012). But compared to animals, AS in plants occurs mainly during stress – with intron retention being the most common event (Mastrangelo et al., 2012; Kornblihtt et al., 2013; Martín et al., 2021). A still unsettled question is, to which extent alternatively spliced mRNA species contribute to protein diversity (Chaudhary et al., 2019) – also under normal growth conditions, and in this context – among Arabidopsis isoforms that catalyze the irreversible OPPP reactions. We wondered whether the possibility to generate novel proteoforms by AS may also change the sites where NADPH is formed, e.g., by targeting OPPP enzymes to different subcellular locations, which has been largely neglected so far.

Here, we show that among the annotated OPPP isoforms of *A. thaliana* (Kruger and von Schaewen, 2003), some may differ at their N- or C-terminal ends. The data obtained for G6PD5 (vs. G6PD6), PGL2, and PGD2 are based on transient expression of fluorescent reporter fusions in Arabidopsis mesophyll protoplasts. This allowed for co-expression with organelle-specific markers and functionality tests using G6PDH-deficient cells. Alternative splicing of *G6PD5* (but not *G6PD6*) resulted in two different, N-terminally extended membrane-bound proteoforms. Together with PGL2 (containing a C-terminal CaaX motif for prenylation), and palmitoylated peptides detected for all three PGD isoforms in an Arabidopsis palmitoylome (Kumar et al., 2020), our results demonstrate that the OPPP may operate as metabolon at the cytosolic face of the ER, where several important enzyme assemblies of higher plant primary and secondary metabolism rely on NADPH provision; for example, the elongation of fatty acids to very long-chain fatty acids (VLCFAs, C_20_-C_24_) as precursors of sphingolipids, triacylglycerols, cutins, and waxes (reviewed in Haslam and Kunst, 2013), or the biosynthesis of phenylpropanes leading to lignin precursors or colored compounds that start with cinnamate 4-hydroxylase (reviewed by Winkel, 2004; Jørgensen et al., 2005). Besides other important plant compounds (such as glucosinolates or thalianol in Arabidopsis), also the biosynthesis of phytohormones may take place at the ER. In fact, indole-3-acetic acid (IAA) can be synthesized from tryptophan in two steps, *via* TAR (tryptophan aminotransferase) and YUCCA enzymes (NADPH-dependent), with the YUC4.2 splice variant carrying a tail anchor for ER insertion (Kriechbaumer et al., 2012). We therefore extended our analyses to KCR1 (ketoacyl-CoA reductase component of fatty acid elongase complex), ATR1 as NADPH:cytochrome P450 oxidoreductase (Laursen et al., 2021) and C4H/CYP73A5, since cytochrome P450 (CYP) enzymes depend on reduced NADPH-hemoprotein reductases (ATR) and thus indirectly on NADPH provision at the ER.

## Materials and methods

### Bioinformatics

Information on *A. thaliana* was retrieved from ARAMEMNON (http://aramemnon.uni-koeln.de/) and The Arabidopsis Information Resource (TAIR; https://www.arabidopsis.org/). Bioinformatic predictions on subcellular protein localization were obtained from DeepLoc-1.0 (https://services.healthtech.dtu.dk/service.php?DeepLoc-1.0) and predictions on transmembrane domains and/or signal peptides from Phobius (https://phobius.sbc.su.se/). Analyses on nucleotide and protein sequences were done with Clustal Omega (https://www.ebi.ac.uk/Tools/msa/clustalo/) provided by the EMBL's European Bioinformatics Institute (Heidelberg, Germany) or the Swiss Institute of Bioinformatics using the Expasy Translate tool (https://web.expasy.org/translate/).

### Arabidopsis mutants

Double-mutant plants lacking cytosolic G6PDH activity were kindly provided by Claudia Jonak (Vienna, Austria). They were obtained by crossing single-mutant plants of SALK_0450839 and GABI-KAT_142G07. In the latter, the T-DNA is inserted 5′ of the regulatory phosphorylation site (Dal Santo et al., 2012). Therefore, the *g6pd5-1 g6pd6-2* double mutant used in this study differs from the one used by Wakao et al. (2008), namely, *g6pd5-1 g6pd6-1* (SALK_016157).

### RT-PCR analyses

Total RNA species were isolated from 8-day-old light-grown *A. thaliana* wild-type (Col-0) seedlings using the NucleoSpin RNA Plant Kit (Macherey-Nagel). Reverse transcription (RT) of mRNA to cDNA was performed with Superscript II Reverse Transcriptase (Invitrogen) and Oligo(dT)_18_ primers (ThermoScientific) after DNA removal with RNase-free DNase I (Invitrogen). PCR amplification from cDNA was conducted with 0.1–1 μl of the RT reaction, the appropriate primer combinations (10 μm each), and Taq DNA polymerase (Biozym). For semi-quantitative RT-PCR analyses, a limited number of cycles were conducted with a 1:5 diluted cDNA template, removing samples at the indicated cycle (72°C step) and transfer to another thermocycler for completing the final elongation step at 72°C for 5 min (and then kept on ice). Samples of the same primer combination (cDNA from seedlings grown on 0 or 1% sucrose) were run on the same gel and documented side-by-side with Molecular Imager^®^ Gel Doc™ XR+ (BIO-RAD, Munich) using the Image Lab™ software optimized for faint band detection.

### Plant growth conditions for tissue culture

Seeds of Arabidopsis wildtype (Col-0) or the cytosolic *G6PD* double mutant (*g6pd5-1 g6pd6-2*) were surface-sterilized. Liquid-suspended seed aliquots were vortexed and incubated for ~2–5 min in different concentrations of ethanol [70, 100, and 70% (v/v)], left to dry on sterile filter paper and placed on a sterile growth medium containing 0.5 × Murashige and Skoog salts with vitamins, pH 5.7, 0.8% agar (w/v), and 1% (w/v) sucrose, if not stated otherwise (0% suc). For stratification, plates were kept at 4°C for at least 2 days and then cultivated under short day conditions (8-h light/22°C, 16-h darkness/20°C) in a growth cabinet. For RT-PCR analyses, seedlings were harvested at the 4-leaf stage and snap frozen in liquid nitrogen. 1-week-old plants were transferred to sterile Magenta boxes (Sigma), containing the same medium (nine seedlings per box), and further cultivated under a short day regime (9-h light/23°C, 15-h darkness/21°C) in a tissue culture room. For protoplast isolation, the rosette leaves of 3- to 4-week-old plants were harvested.

### Cloning of fluorescent reporter fusions

The cloning of all fluorescent reporter fusion constructs was performed as described earlier (Meyer et al., 2011; Hölscher et al., 2014, 2016; Baune et al., 2020; Lansing et al., 2020). Formerly used pGFP-/pOFP2-NX vectors (Hölscher et al., 2016) were modified by site-directed mutagenesis. First, GFP was converted to a monomeric version (*m*GFP) by one amino acid exchange (alanine 206 to lysine, Zacharias et al., 2002). Then, a NotI-restriction site was introduced in front of the 35S promoter to create a second site for insertion (*via* NotI and PstI/SdaI), resulting in vector p*m*GFP-NXn (pos. 4488) used in this study. The NotI site was also introduced into the pOFP2-NX vector (pos. 4449), leading to pOFP-NXn and completed by deletion of the PstI-restriction site in the open reading frame of OFP (orange-shifted *m*RFP). For reporter fusions, open reading frames were amplified from seedling cDNA or existing plasmid constructs and cloned into the plant expression vectors *via* compatible restriction sites. For splice variant *G6PD5.5*, the 5′ region (N-terminus) was amplified from genomic DNA of Arabidopsis Col-0 wildtype and inserted into the plant expression vector containing *G6PD5.4* (already fused to *m*GFP), thus changing the corresponding part of the N-terminus. Split-YFP constructs (G6PD5.4-pSPYNE and pSPYCE(MR)-PGL2) were cloned for bimolecular fluorescence complementation (BiFC) assays essentially as described in Meyer et al. (2011). All constructs were confirmed by sequencing. For the oligonucleotide primers used in this study, refer to Supplementary Table S1.

### Cloning of double cassette constructs

For the reporter-less constructs in a double cassette plasmid, first, the cDNA fragment was cloned behind the reporter in the G/OFP-NX vectors *via* SpeI/BamHI, checked by sequencing, and tested in protoplasts for subcellular localization. Then, the reporter was deleted *via* XbaI and SpeI, followed by relegation of the compatible ends. The resulting reporter-less construct was used as a template to amplify the reporter-less expression cassette (consisting of the 35S promotor, open reading frame of interest, and NOS terminator) using primers 1202 and 1203. The cassette was then inserted *via* NotI and PstI/SdaI into the *m*GFP/OFP-NXn vectors.

### Site-directed mutagenesis

Base changes were introduced into PGL2 by the QuickChange PCR mutagenesis protocol (Stratagene) using appropriate primer combinations (Supplementary Table S1) and S7 Fusion High-Fidelity DNA Polymerase (Biozym). All base changes were confirmed by sequencing.

### Protoplast transfection

Protoplasts were isolated from Arabidopsis leaf mesophyll cells and transfected with plasmid DNA by a PEG-mediated method as described previously (Baune et al., 2020; Lansing et al., 2020). For each sample, ~500,000 protoplasts were mixed with plasmid DNA and dissolved in TE buffer (1 mM EDTA, 10 mM Tris pH 7.5). For co-expression studies, the DNA of both constructs was mixed beforehand and filled up to a volume of 60 μl with TE buffer. Generally, for proteins of interest, 15 μg of plasmid DNA was used. For organelle markers, 2 μg of plasmid DNA was used for the ER marker, 7 μg for the peroxisomal ER marker, and 5 μg for the soluble ER marker. For BiFC analyses, 20 μg of plasmid DNA was used for each construct. After transfection, protoplast samples were incubated in the dark at room temperature and analyzed after ~24–48 h. The expression of all reporter constructs was driven by the CaMV-35S promoter. MitoTracker orange (Molecular Probes, Invitrogen) was used as recommended by the supplier and imaged after 20-min incubation.

### Confocal laser-scanning microscopy

Localization studies were conducted with a confocal laser-scanning microscope (Leica TCS SP5, https://www.leica-microsystems.com/). The following settings for excitation/emission wavelengths were used: 488/490–520 nm for GFP and 561/590–620 nm for OFP (Lansing et al., 2020).

### Ratiometric topology analyses of *ro*GFP fusion proteins

For ratiometric topology analyses, *ro*GFP fusions were cloned as described previously (Baune et al., 2020). Fluorescent images were recorded with a confocal laser-scanning microscope SP5 (Leica, https://www.leica-microsystems.com/) at excitation wavelengths of 405 nm and 488 nm in a sequential scan. Using the colors green and red for the 405-nm scan and the 488-nm scan, respectively, is a quick method to visualize the approximate redox potential. A greenish overlay indicates oxidized conditions (ER lumen), whereas an overlay of red to orange/yellow indicates reduced conditions (cytosol).

### Determination of G6PDH activity

For activity measurements, Arabidopsis mesophyll protoplasts were transfected with fluorescent reporter fusion constructs previously used for localization studies. The empty *m*GFP-Nxn vector served as negative control. To obtain enough protein for both, activity measurements and immunoblot analyses, 3–4 independent transfections were performed (total number of 1.5–2 million protoplasts) and pooled after ~48 h of incubation. Cells were harvested by centrifugation in a 2-ml reaction vial, with the supernatant being discarded, shock-frozen in liquid nitrogen, and stored at −80°C until analyses. Frozen cells were resuspended in 50 μl extraction buffer, containing 100 mM HEPES, pH 7.5, 2 mM Na_2_S_2_O_5_, 5 μl protease inhibitor cocktail (for use with plant extracts, Sigma), and 0.02% Triton X-100. The G6PDH activity for each sample was determined as described previously (Meyer et al., 2011).

### GFP-trap enrichment

Arabidopsis protoplasts were transfected as described above. Different from the setup for localization studies, one million protoplasts were transfected with 60 μg of plasmid DNA. After 48-h incubation at room temperature in the dark, 10 independent transfections were pooled. For immunoprecipitation, ChromoTek GFP-Trap^®^ Agarose beads (ChromoTek, Munich) were used following the recommended protocol. Our lysis buffer contained 50 mM Tris-HCl, pH 7.5, 250 μm NADP^+^ (for G6PD stabilization) 5% (v/v) glycerol, 0.5 μl/ml β-mercaptoethanol, 150 mM NaCl, 0.1% Triton X-100, 1 mM Pefabloc SC, and 1% protease inhibitor cocktail (for use with plant extracts, Sigma). The dilution buffer was the same, but without β-mercaptoethanol, Triton X-100, and protease inhibitor cocktail. As wash buffer, we chose 50 mM Tris-HCl, pH 7.5, 250 μm NADP^+^ (for G6PD stabilization), 1% glycerol, 150 mM NaCl, and 0.05% Triton X-100. Different from the original protocol, beads were washed two times and then resuspended in 100 μl buffer containing 50 mM Tris-HCl, pH 7.5, 250 μm NADP^+^ (for G6PD stabilization), 1% glycerol, and 1 mM MgCl_2_. Aliquots were used for SDS-PAGE followed by immunoblot analyses and frozen samples for mass spectrometry analyses (in-house proteomic platform).

### Immunoblot analyses

Generally, immunoblot analyses were performed as described previously (Hölscher et al., 2016; Baune et al., 2020; Lansing et al., 2020). As variation, blocking of the blots was conducted with 5% (w/v) milk powder in TBST (TBS with 0.05% (v/v) Tween-20) instead of 2%, which resulted in less background. Blot stripping was conducted as described in the study by Lansing et al. (2020).

### Mass spectrometry-based quantitative proteomics

Proteins were cleaned up and digested following the SP3 protocol (Hughes et al., 2019). An LC-MS/MS analysis was performed using an EASY-nLC 1200 (ThermoFisher) coupled with an Exploris 480 mass spectrometer (ThermoFisher). Separation of peptides was performed on 25-cm frit-less silica emitters (CoAnn Technologies, 0.75-μm inner diameter), packed in-house with reversed-phase ReproSil-Pur C_18_ AQ 1.9 μm resin (Dr. Maisch). The column was constantly kept at 50°C. Peptides were eluted for 115 min applying a segmented linear gradient of 0–98% solvent B (solvent A 0% ACN, 0.1% FA; solvent B 80% ACN, 0.1% FA) at a flow rate of 300 nl/min. Mass spectra were acquired in data-dependent acquisition mode. In the case of full proteome samples, MS^1^ scans were acquired at an Orbitrap resolution of 120,000 with a scan range (*m*/*z*) of 380–1,500, a maximum injection time of 100 ms, and a normalized AGC target of 300%. For fragmentation, only precursors with charge states 2–6 were considered. Up to 20 dependent scans were taken. For dynamic exclusion, the exclusion duration was set to 40 s and a mass tolerance of ± 10 ppm. The isolation window was set to 1.6 *m*/*z* with no offset. A normalized collision energy of 30 was used. MS^2^ scans were taken at an Orbitrap resolution of 15,000, with a fixed first mass (*m*/*z*) = 120. The maximum injection time was 22 ms, and the normalized AGC target was 50%.

Processing of raw data was performed using the MaxQuant software version 2.0.3.0 (Tyanova et al., 2016). MS/MS spectra were assigned to the Araport11 protein database. During the search, sequences of 248 common contaminant proteins as well as decoy sequences were automatically added. Trypsin specificity was required and a maximum of two missed cleavages was allowed. Carbamidomethylation of cysteine residues was set as fixed, oxidation of methionine, and protein N-terminal acetylation as variable modifications. A false discovery rate of 1% for peptide spectrum matches and proteins was applied. Matches between runs and iBAQ were enabled.

### Data availability

For review purposes, the MS raw data are available *via* the following credentials and will be made freely available *via* the JPOST repository (Okuda et al., 2017) under the identifier JPST001685.

URL: https://repository.jpostdb.org/preview/125256832562cc3a65952c7.

Access key: 7446.

## Results

### Alternative splice variants of *G6PD5* encode N-terminally extended proteoforms

**Figure 1:**
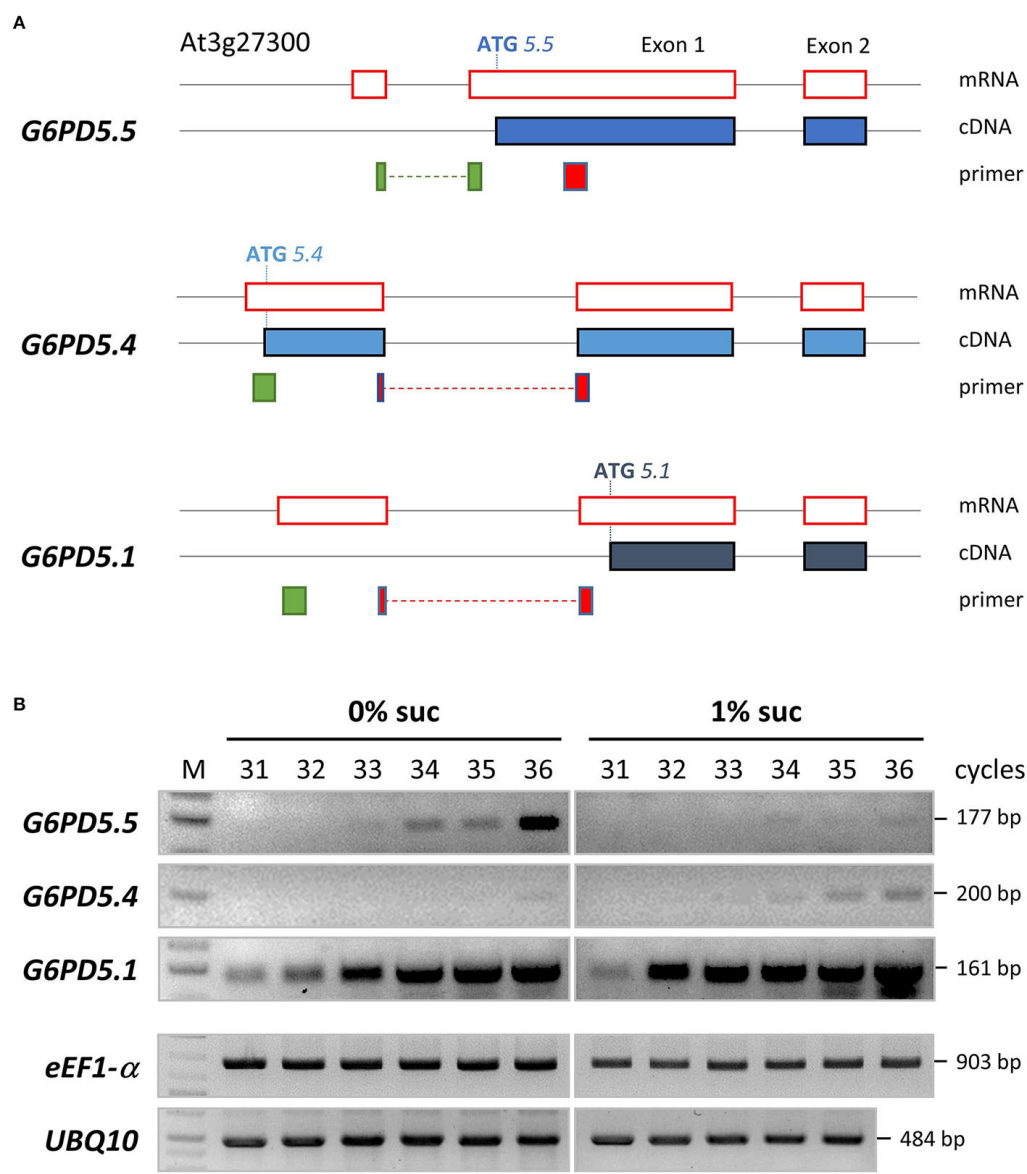
Alternative splice variants and semi-quantitative RT-PCR analyses of *G6PD5*. **(A)** To scale representation of the *G6PD5* locus (At3g27300) with 5′ UTR until exon 2. Black lines, genomic DNA; red boxes, mRNA parts upon splicing; filled blue boxes, resulting coding sequences (cDNA). Note that alternative splicing leads to different ATG start positions. Primer binding sites are depicted underneath (green, sense; red, antisense primers, with intron-spanning regions as dashed lines). According to an RNA-seq tool (Arabidopsis.org, ARAPORT11/splice junctions and/transcript assembly), *G6PD5.1* and *G6PD5.4* are the most abundant splice variants, whereas *G6PD5.5* is not present in all tissues, but amounts to about 10% of the other two (e.g., in light-grown seedlings, 476 counts for *G6PD5.4* and *G6PD5.1* compared to 52 for *G6PD5.5*). **(B)** Semi-quantitative RT-PCR analyses of RNA isolated from seedlings (4-leaf stage) grown under a short-day regime in the absence (0% suc) or presence of sucrose (1% suc) on agar plates (0.8%, 0.5 MS, pH 5.7). Note that *G6PD5.5* is more abundant under autotrophic conditions (0% suc), whereas *G6PD5.4* (and by trend also *G6PD5.1*) is more abundant under the physiological sink condition (1% suc). Two house-keeping genes, e*EF1-*α (eukaryotic translation elongation factor, At5g60390) and *UBQ10* (polyubiquitin10, At4g05320), were included as reference. M, GeneRuler 1kb Plus DNA Ladder (ThermoFisher Scientific).

Our work started with the notion that several splice variants are listed for the two cytosolic Arabidopsis isoforms *G6PD5* and *G6PD6*, but only *G6PD5.4* and *G6PD5.5* may encode novel proteoforms with extended N-terminal ends (Figure 1A, ATG start sites). RT-PCR analyses confirmed the presence of the three splice forms in Arabidopsis seedlings, and semi-quantitative RT-PCR analyses revealed that the *G6PD5.5* variant is more abundant under photoautotrophic conditions (0% sucrose) as compared to *G6PD5.4* and *G6PD5.1* that were more abundant under heterotrophic conditions (1% sucrose; Figure 1B).

**Figure 2:**
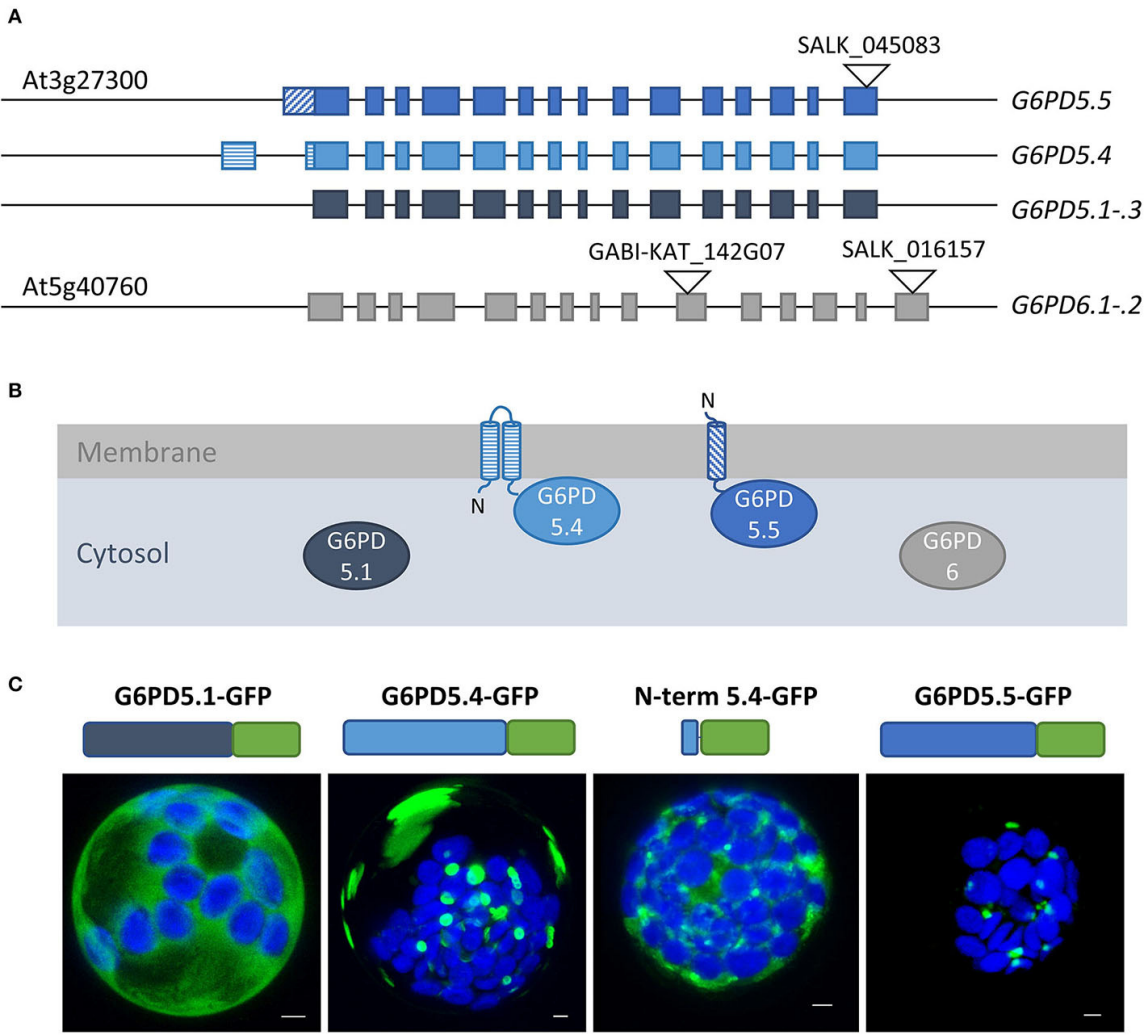
Alternative splice variants of *G6PD5* encode N-terminally extended proteoforms. **(A)** To-scale depiction of the splice variants that may be generated from the cytosolic *G6PD* loci, five from *G6PD5* (At3g27300) and two from *G6PD6* (At5g40760). Compared to splice variants *G6PD5.1* through *.3* (and *G6PD6.1-.2*) *G6PD5.4* and *.5* code for N-terminally extended proteoforms. Exons are shown as boxes with alternative 5′ parts in different patterns. T-DNA insertion positions in the indicated mutant lines are depicted as black triangles (GABI-KAT_142G07 was used for obtaining the *g6pd5-1 g6pd6-2* double mutant used in this work). **(B)** Membrane topology of proteoforms G6PD5.4 and G6PD5.5 according to hydropathy plots and bioinformatic predictions (for details, refer to Figure 3A and Supplementary Figure S1). **(C)** Localization patterns of the different G6PD5 proteoforms with C-terminally fused GFP (schematically depicted above) in Arabidopsis wild-type protoplasts. Next to local accumulations, the G6PD5.4-GFP versions (full-length or the N-terminal part) labeled also patchy areas that were missing from G6PD5.5-GFP. The images show maximal projections of about 30 single optical sections. GFP in green and chlorophyll autofluorescence in blue. Scale bars, 3 μm.

Bioinformatic tools predicted two membrane-spanning domains for G6PD5.4 and one for G6PD5.5 (Supplementary Figure S1A), resulting in different membrane topologies (Figures 2A,B). Subcellular localization prediction for G6PD5.4 was the endoplasmic reticulum (ER) and for G6PD5.5, it was the cytosol (Supplementary Figure S1B). Therefore, proteoform-specific primers (Figure 1A) were used for cloning reporter fusions, first with C-terminal monomeric GFP. Indeed, compared to cytosolic G6PD5.1-GFP, G6PD5.4-GFP and G6PD5.5-GFP localized to distinct cellular sites (Figure 2C), with only the N-terminal domain of G6PD5.4 fused to GFP still showing local accumulations.

**Figure 3:**
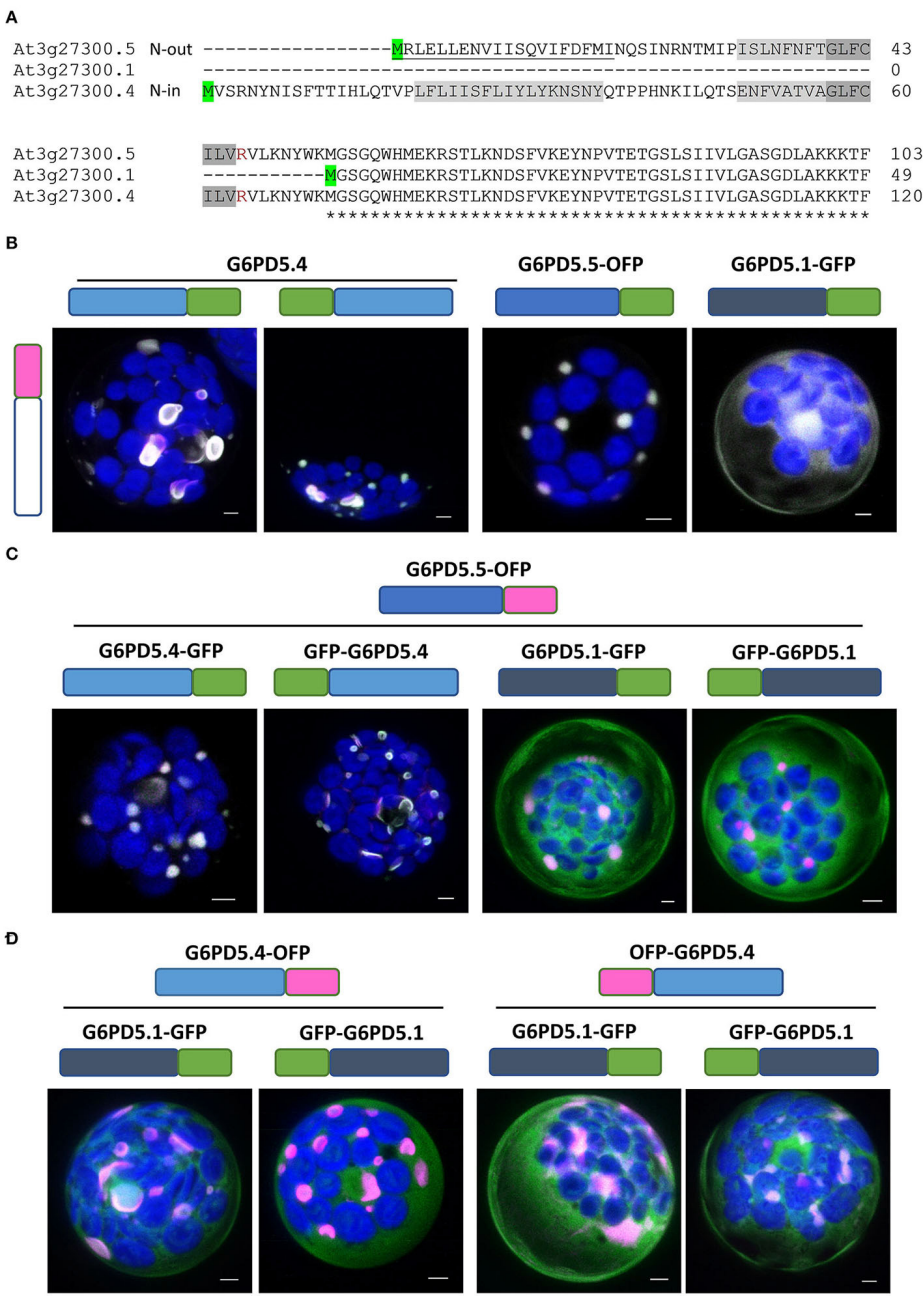
Extent of co-localization among the same or different G6PD5 proteoforms. **(A)** N-terminal part of a sequence alignment (CLUSTAL Omega) of the three G6PD5 proteoforms. Start codons are highlighted in green. Relative to the transmembrane regions (shaded gray, identical parts dark gray), the N-terminus of G6PD5.5 is predicted by ARAMEMNON as N-out (signal peptide, underlined) and of G6PD5.4 as N-in (cytosolic). **(B)** Homotypic co-expression of the same proteoform (open symbol); **(C,D)** heterotypic co-expression of different proteoforms. If not indicated, the images show maximal projections of about 30 optical sections as a merger of all channels (for single channel images, refer to Supplementary Figure S2). GFP in green, OFP in magenta, and chlorophyll autofluorescence in blue; white signals indicate co-localization of GFP and OFP (or very close signals < 200 nm). Scale bars, 3 μm.

For further analyses, reporter fusions with OFP (orange-shifted *m*RFP) were cloned as well. First, the extent of co-localization was tested among the same and different G6PD5 proteoforms. Since the topology prediction of Phobius (https://phobius.sbc.su.se/) for G6PD5.4 was “N-in” and for G6PD5.5 “N-out” (Figure 3A; Supplementary Figure S1A), N-terminally tagged reporter fusions were also cloned for the G6PD5.4 proteoform (with 16 amino acid spacer). Homotypic co-expression of oppositely labeled reporter fusions (using the same proteoform) showed co-localization for all G6PD5 proteoforms (white signals, Figure 3B; for single channel images, refer to Supplementary Figure S2). This was also seen for co-expression of the two N-terminally extended proteoforms (Figure 3C, left images), but much less when G6PD5.5 (Figure 3C, right images) or G6PD5.4 (Figure 3D) was co-expressed with the cytosolic G6PD5.1-reporter fusions.

### The G6PD5.4 and G6PD5.5 proteoforms localize to different subdomains of the ER

**Figure 4:**
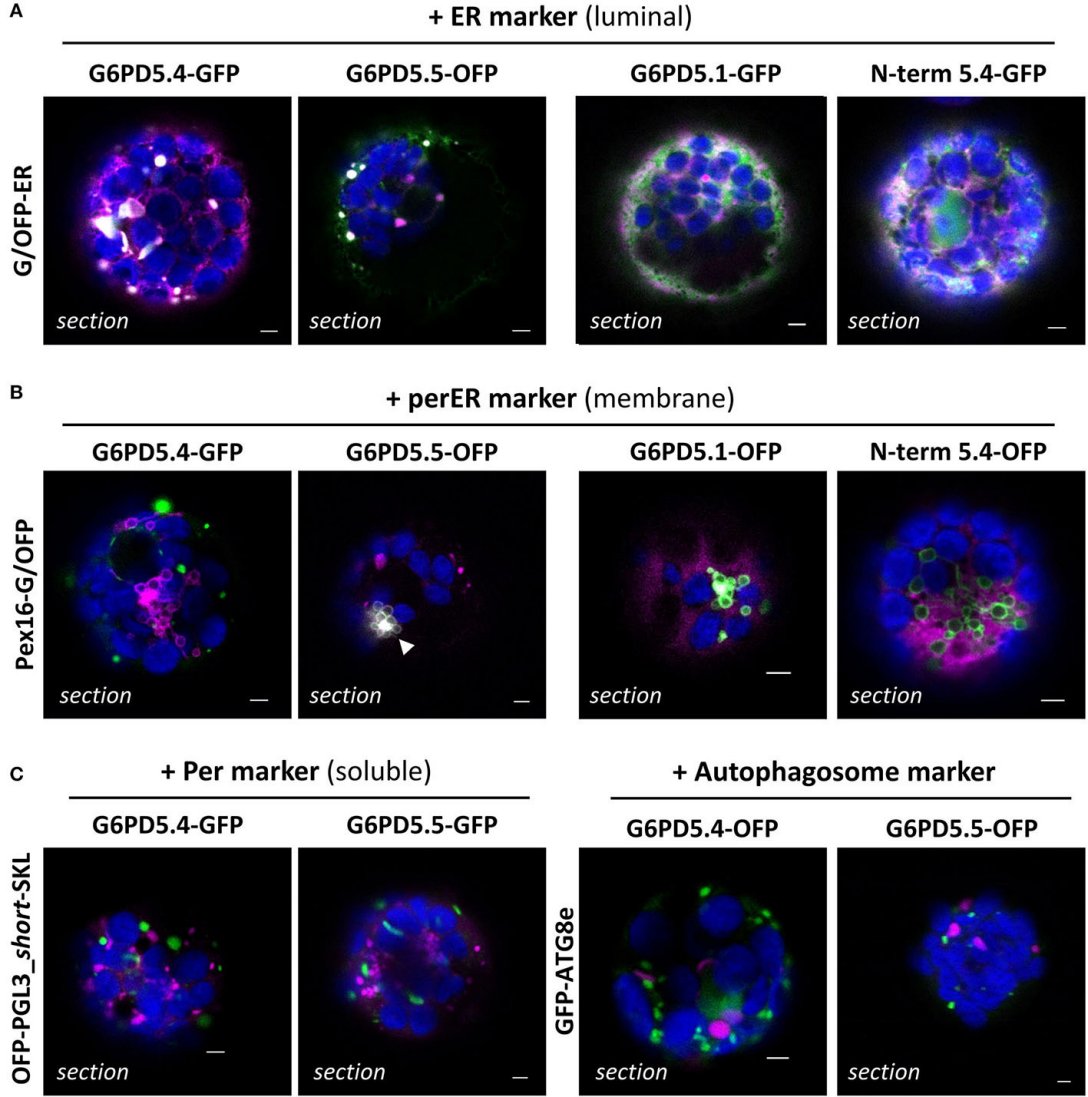
Subcellular localization of the membrane-bound G6PD5.4 and G6PD5.5 reporter fusions. **(A)** Co-expression of the indicated G6PD5-reporter fusions with a luminal ER marker (GFP-ER or OFP-ER) showed local overlap with G6PD5.4 and G6PD5.5 (white signals), little with G6PD5.1, and some with the N-terminal G6PD5.4 part. **(B)** G6PD5.4 showed no overlap with Pex16 (marker of the peroxisomal ER), but G6PD5.5 did in some areas (white arrowhead). **(C)** Despite accumulating in spherical structures reminiscent of peroxisomes, neither G6PD5.4 nor G6PD5.5 co-localized with a soluble peroxisome marker (PGL3_*C-short*-SKL) or an autophagosome marker (ATG8e). The images show single optical sections as a merger of all channels (for single channel images, refer to Supplementary Figure S3). GFP in green, OFP in magenta, and chlorophyll autofluorescence in blue; white signals indicate co-localization of GFP and OFP (or very close signals < 200 nm). Scale bars, 3 μm.

To determine the exact subcellular localization of the alternative G6PD5 proteoforms, the GFP- and OFP-based reporter fusions were used for co-expression with oppositely labeled organelle markers. G6PD5.4 and G6PD5.5 clearly labeled subdomains of the ER (Figure 4A, white signals), which was not the case for G6PD5.1 and less with the N-term_G6PD5.4 construct (for single channel images, refer to Supplementary Figure S3). Although showing overlap with the soluble ER marker, local accumulations of G6PD5.4 and G6PD5.5 localized to different ER sites (Figure 4B). Neither full-length G6PD5.4 nor the N-term_5.4 fusion co-localized with Pex16 (at the peroxisomal ER), yet G6PD5.5 did (white signals). Because the local ER accumulations resembled peroxisomes in size, additional co-expressions were conducted with a soluble peroxisome and also with an autophagosome marker; however, both did not show any overlap (Figure 4C).

### The catalytic domains of G6PD5.4 and G6PD5.5 face the cytosol and are functional

**Figure 5:**
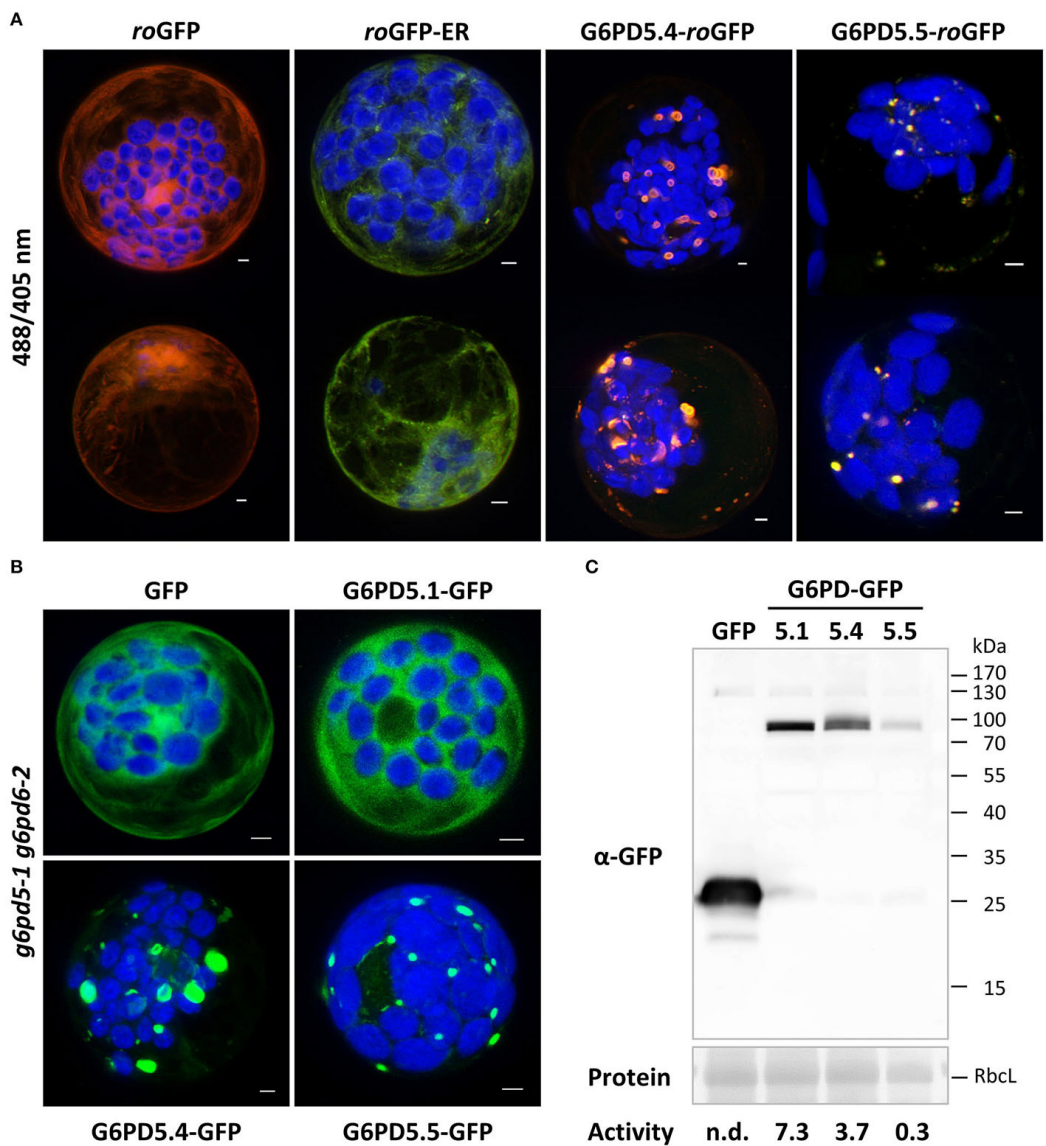
Topology and functionality of the membrane-bound G6PD5.4 and G6PD5.5 proteoforms. **(A)** Redox-sensitive GFP (*ro*GFP) was used to demonstrate that the catalytic domains of G6PD5.4 and G6PD5.5 face the cytosol. Two representative cells are shown for each construct. Note that the 488/405 ratios resemble cytosolic *ro*GFP (red-orange/yellow) and not luminal *ro*GFP-ER (green). For single channel images, refer to Supplementary Figure S4. **(B)** Protoplasts were prepared from *g6pd5-1 g6pd6-2* double-mutant plants (lacking cytosolic G6PDH activity) and transfected with the indicated G6PD5-GFP fusions. The images show maximal projections of about 30 optical sections as a merger of both channels. GFP in green, chlorophyll autofluorescence in blue. Scale bars, 3 μm. **(C)** Immunoblot analysis of protoplast extracts (separated on a 12% SDS gel) using anti-GFP antibodies (α-GFP). Protein, Ponceau S-stained blot, with RbcL (RubisCO, Large subunit) as loading reference. Molecular mass standard (in kDa), PageRuler Prestained Protein Ladder (Fermentas). Activity, volume equivalents of NADPH formed (nmol/min/ml), which was not detectable (n.d.) for the control (GFP). Note that enzymatic activity of the G6PD5-GFP fusions (bottom) corresponds to the intensity of their respective blot signals, indicating that the membrane-bound proteoforms form active homomers at the ER.

Membrane topology at the ER can be analyzed by taking advantage of differences in redox state between the ER lumen (largely oxidized) compared to the cytosol (largely reduced). To confirm that the catalytic domains of the ER-bound G6PD5 proteoforms face the cytosol, redox-sensitive GFP (*ro*GFP) was fused to the C-terminal end of G6PD5.4 and G6PD5.5. The resulting constructs were analyzed in parallel to the cytosolic *ro*GFP and luminal *ro*GFP-ER controls (Figure 5A). Again, local accumulations were detected for G6PD5.4-*ro*GFP and G6PD5.5-*ro*GFP. False color images of the 488/405 nm ratios resembled the cytosolic *ro*GFP control (orange-to-red) rather than the luminal *ro*GFP-ER version (yellow-to-green; for single channel images, refer to Supplementary Figure S4). To test for functionality of the membrane-bound catalytic domains, mesophyll protoplasts were prepared from *g6pd5-1 g6pd6-2* double-mutant plants lacking cytosolic G6PDH activity (similar to the *g6pd5-1 g6pd6-1* combination used by Wakao et al., 2008) and transfected with the indicated G6PD5-GFP fusions (Figure 5B). Protoplasts were harvested and extracted for determining G6PDH activity and immunoblot analyses in parallel (Figure 5C). Compared to GFP expressed in the *g6pd5-1 g6pd6-2* double mutant (for which G6PDH activity was undetectable), G6PD5.1-GFP was about two times as active as G6PD5.4-GFP. G6PD5.5-GFP showed only one-tenth activity of G6PD5.4-GFP, yet these values corresponded well to the signal strengths at about 90 kDa. Therefore, both membrane-bound G6PD5 proteoforms appear to be active at a comparable level – notably as homomers.

### G6PD5.4 and the 2nd OPPP enzyme PGL2 co-localize in ER subdomains

**Figure 6:**
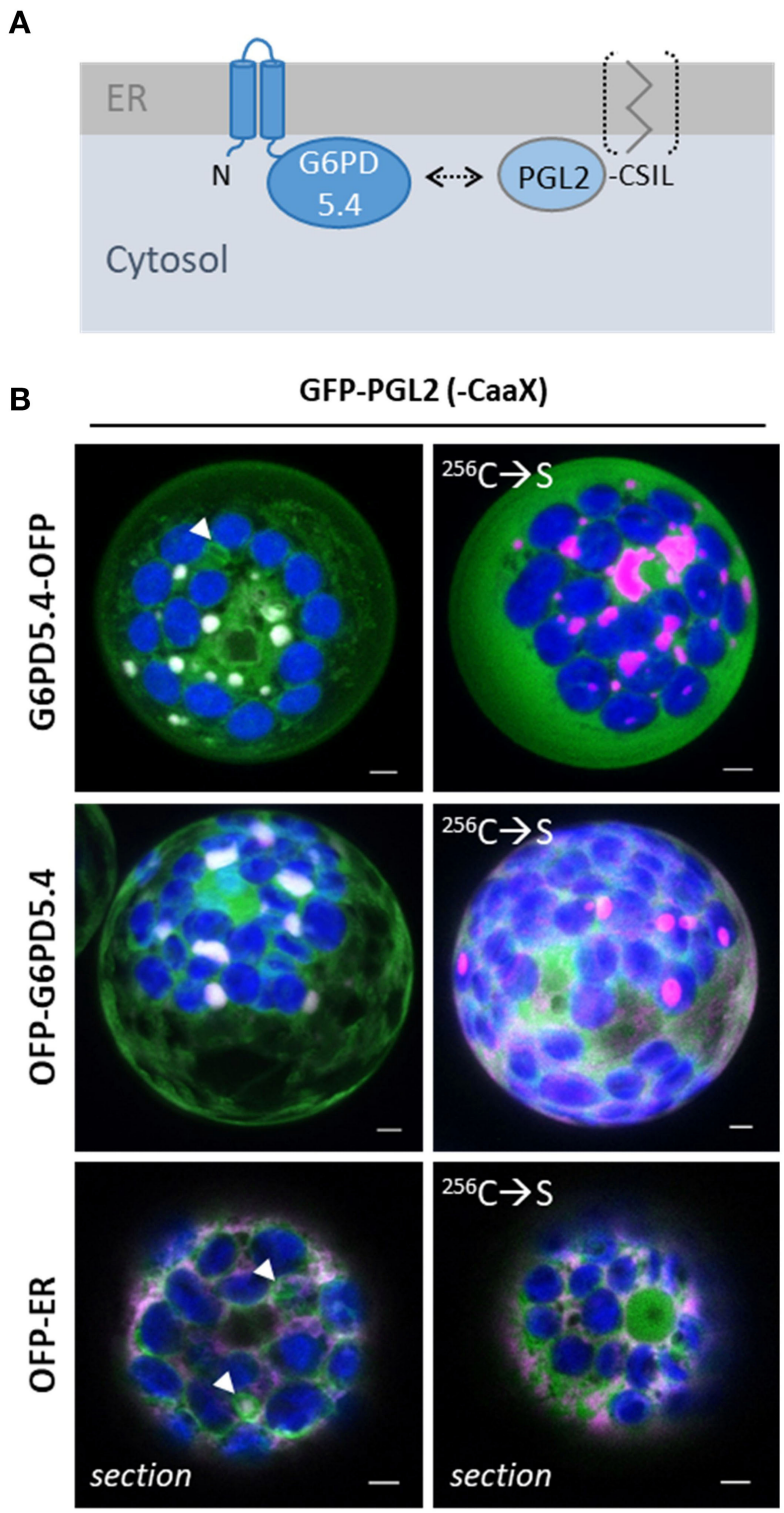
G6PD5.4 recruits PGL2 with C-terminal CaaX motif to ER subdomains. **(A)** Scheme depicting that G6PD and PGL isoforms may interact (arrow) to avoid the release of dead-end byproduct χ6-phosphogluconolactone (Miclet et al., 2001). **(B)** GFP-PGL2 (with C-terminal CaaX motif that may be prenylated for membrane association) was co-expressed with G6PD5.4-OFP or OFP-G6PD5.4 in Arabidopsis wild-type protoplasts. Note that the mutated ^256^C → S version (destroyed CaaX motif) labels the cytosol (and nucleus, bottom right). Co-expression with a soluble ER marker (OFP-ER) demonstrated that on its own, also PGL2 tends to accumulate in distinct membrane regions (white arrowheads). If not indicated, the images show maximal projections of about 30 optical sections as a merger of all channels (for single channel images, refer to Supplementary Figure S5). GFP in green, OFP in magenta, and chlorophyll auto-fluorescence in blue; white signals indicate co-localization of GFP and OFP (or very close signals < 200 nm). Scale bars, 3 μm.

During the previous analysis of the five PGL isoforms (Lansing et al., 2020), PGL2 was occasionally found around plastids. The Aramemnon server (http://aramemnon.uni-koeln.de/index.ep) predicted lipid modification *via* a C-terminal CaaX motif (-CSIL, Figure 6A). Thus, a full-length GFP-PGL2 construct was cloned and co-expressed with the full-length OFP fusion constructs of G6PD5.4. Co-localization was observed in local patches, which was revoked by Cys-to-Ser mutation in the C-terminal CaaX motif of PGL2 (Figure 6B). Thus, loss of membrane attachment (Figure 6A, bracket) resulted in the labeling of the cytosol and the nucleus. Note that in co-expression with G6PD5.4 and the ER marker, PGL2 with intact CaaX motif resides in ER structures that appear to represent extra membranous material (Figure 6B, white arrowheads; for single channel images, refer to Supplementary Figure S5). Apparently, only membrane-bound PGL2 (as the second OPPP enzyme) is recruited by G6PD5.4 to ER subdomains.

### G6PD5.4 localizes in ER subdomains independent of PGD2 or PGL2

**Figure 7:**
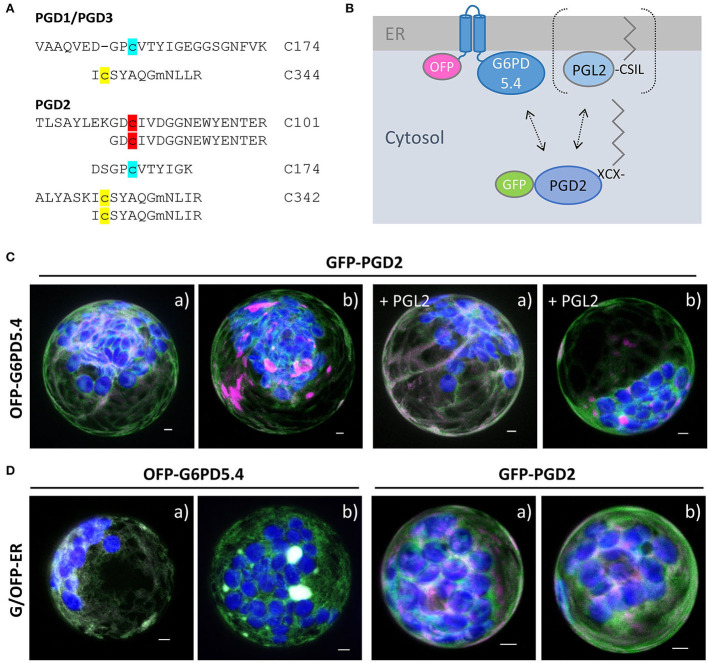
Co-expression of OFP-G6PD5.4 and GFP-PGD2 without or with PGL2. 6-phosphogluconate dehydrogenase (PGD) is the second OPPP enzyme contributing to NADPH provision. **(A)** PGD peptides found in an Arabidopsis palmitoylome for PGD1/3 (cannot be discriminated) and PGD2 (Kumar et al., 2020). Cysteine positions shared by all PGD isoforms are labeled in yellow/blue, and the unique one of PGD2 in red. Among reporter fusions of the three PGD isoforms, PGD2 showed weak co-localization with PGL2 at the ER (Supplemental Figure S6). **(B)** To test whether PGL2 might function as a bridge between the two OPPP dehydrogenases, the GFP reporter was deleted and the resulting expression cassette with “naked” PGL2 was inserted into the OFP-G6PD5.4 construct (Nxn site). **(C)** Co-expression of GFP-PGD2 with OFP-G6PD5.4 either without or with PGL2 (+PGL2). **(D)** OFP-G6PD5.4 or GFP-PGD2 were co-expressed with the luminal ER marker (GFP-ER or OFP-ER) as control. Note that two different patterns (a, b) were observed. The images show maximal projections of about 30 optical sections as a merger of all channels (for single channel images, refer to Supplemental Figure S7). GFP in green, OFP in magenta, and chlorophyll autofluorescence in blue; white signals indicate co-localization of GFP and OFP (or very close signals < 200 nm). Scale bars, 3 μm.

For one of the three Arabidopsis PGD isoforms that catalyze the third OPPP step and show a high degree of homology (Hölscher et al., 2016), palmitoylation was experimentally shown for PGD3 (At5g41670) by Hemsley et al. (2013). However, in a recently published Arabidopsis palmitoylome (Kumar et al., 2020), palmitoylated peptides were detected for all PGD isoforms: the same two for PGD1 (At1g64190) and PGD3 (At5g41670; Figure 7A, modified cysteines in yellow and blue), and an additional one for PGD2 (At3g02360; Figure 7A, red). We therefore chose PGD2 for co-localization analyses with OFP-G6PD5.4, leaving the catalytic domain free for interaction. This appeared necessary because G6PD5.4-OFP did not co-localize with GFP-PGD2 (Supplementary Figure S6A), but OFP-PGD2 showed weak overlap with GFP-PGL2 (Supplementary Figure S6B). To investigate a potential influence of co-expressed PGL2 with a free N-terminal end (Figure 7B, bracket), the reporter was deleted from the GFP-PGL2 construct, and the resulting expression cassette was introduced in front of that of OFP-G6PD5.4 (in the NXn vector, refer to Materials and methods). Then, GFP-PGD2 was co-expressed with OFP-G6PD5.4 or OFP-G6PD5.4 harboring unlabeled PGL2 (+PGL2, Figure 7C). OFP-G6PD5.4 accumulated evenly at the ER but also in focal ER sites, independent of the additional presence of PGL2 (for single channel images, refer to Supplementary Figure S7). Co-expression with a soluble ER marker (G/OFP-ER; Figure 7D) also suggests that accumulation in ER subdomains is a feature of G6PD5.4 and largely independent of the presence of PGD2 or PGL2.

### G6PD5.4 is able to establish an OPPP metabolon in ER subdomains

**Figure 8:**
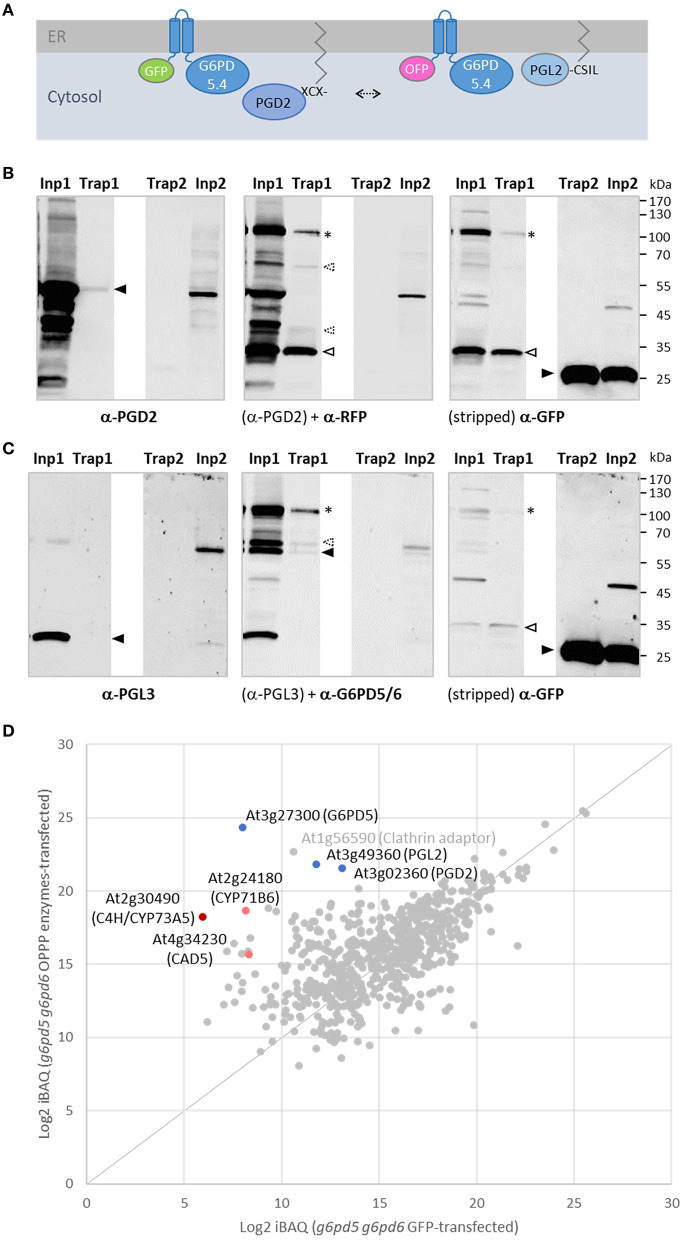
GFP-based pull-down of G6PD5.4 from cells co-expressing label-free OPPP isoforms PGD2 and PGL2. To test whether also PGD2 may associate with G6PD5.4, a reporter-less expression cassette (“naked” PGD2) was introduced into the GFP-G6PD5.4 construct and co-expressed with the OFP-G6PD5.4 construct harboring reporter-less PGL2 (Figure 7). **(A)**, Topology of the protein combinations at the cytosolic face of the ER, leaving the catalytic domain of G6PD5 free for protein-protein interaction (arrow). **(B,C)** Immunoblot analyses of *Arabidopsis g5pd5-1 g6pd6-2* mutant protoplasts co-expressing GFP-G6PD5.4 (+PGD2) and OFP-G6PD5.4 (+PGL2; number 1 samples), or GFP only (control; number 2 samples). Inp, input (protoplast extract); Trap, GFP-Trap affinity resin (ChromoTek) used for pull-down. **(B)** One blot (of two identically loaded 10% SDS gels) was first developed with anti-PGD2 antibodies, followed by anti-RFP antibodies, and after stripping with anti-GFP antibodies. **(C)** The other blot was first developed with anti-PGL3 antibodies, followed by anti-G6PD5/6 antibodies, and after stripping with anti-GFP antibodies. Note that both reporters were partially cleaved from the N-terminally tagged G6PD5.4 proteoforms (asterisks mark bands of the intact fusion proteins, open arrowheads degradation products, and filled arrowheads the cognate antigens. G6PD5.4 was also detected by an antiserum raised against His-G6PD5 and His-G6PD6 (1:1, purified from *Escherichiea coli*), unlabeled PGD2 by the one raised against His-PGD2 (α-PGD, Hölscher et al., 2016), whereas PGL2 was not recognized by antibodies raised against His-PGL3 (Hölscher et al., 2014), possibly due to in-planta prenylation of the C-terminal CaaX motif (compared to unmodified His-PGL2 purified from *E. coli*; Lansing et al., 2020). Strong bands in the input confirmed that unlabeled PGD2 and PGL2 were faithfully expressed. Note the weak PGD2 signal in the Trap1 sample [**(B)**, left] corresponds to a similarly weak GFP signal of GFP-G6PD5.4 [**(B)**, right], indicating a successful pull-down of PGD2. Molecular mass standards (in kDa) are shown on the right (PageRuler Prestained Protein Ladder, Fermentas). **(D)** Quantitative mass spectrometry results of the GFP-G6PD5.4 pull-down from *g6pd5-1 g6pd6-2* mutant protoplasts. The abundance (Log_2_ iBAQ) of proteins pulled from cells transfected with plasmids of the OPPP enzyme combination was plotted against those pulled from cells transfected with GFP only (control). Note that PGL2 was enriched similarly to PGD2. Other proteins pulled-down by GFP-G6PD5.4 were C4H/CYP73A5 (cinnamate-4 hydroxylase); CAD5 (cinnamyl-alcohol dehydrogenase) and CYP71B6, a cytochrome P450 enzyme involved in the biosynthesis of multiple defense compounds in Arabidopsis (Böttcher et al., 2014). Dots representing the OPPP enzymes in blue and of directly (CAD5) or indirectly NADPH-dependent enzymes in red. For the complete list of proteins (gray), refer to Supplementary Table S2.

To investigate whether G6PD5.4 (1st OPPP step) with a free C-terminal end may recruit both PGL2 (2nd OPPP step) and PGD2 (3rd OPPP step) to a membrane-bound metabolon, a reporter-less expression cassette was also prepared for PGD2 and inserted in front of the GFP-G6PD5.4 cassette (NXn site). Co-expression of GFP-G6PD5.4 (+PGD2) with OFP-G6PD5.4 (+PGL2; Figure 8A) in Arabidopsis protoplasts resulted in exclusive co-localization in ER subdomains (similar to the pattern in Figure 3B), likely facilitated by about two-fold higher G6PD5.4 amounts compared to PGL2 and PGD2. Transfected protoplasts of both wild-type and the *g6pd5-1 g6pd6-2* double mutant were harvested and subjected to pull-down with GFP-Trap agarose beads, followed by immunoblot and mass spectrometry analyses. We had already noticed that the GFP reporter tends to be released from GFP-G6PD5.1 and GFP-G6PD5.4 (not shown). Thus, compared to the input (protoplast extract), only a faint band of the full-length GFP-G6PD5.4 fusion protein was recovered (Figure 8B, α-GFP blot, Trap1, marked by an asterisk). Based on the immunoblot results, PGL2 was not co-purified (Figure 8C, α-PGL3 blot, Trap1, missing band marked by a closed arrowhead). However, mass spectrometry analyses showed an even slightly higher enrichment of PGL2 compared to PGD2 (Figure 8D), indicative of physical interaction. Together with the fact that PGL2 was recruited to ER subdomains (Figure 6), G6PD5.4 seems able to establish a complete membrane-bound OPPP metabolon at the cytosolic face of the ER.

### The G6PD5 proteoforms may recruit NADPH-dependent enzymes to ER subdomains

**Figure 9:**
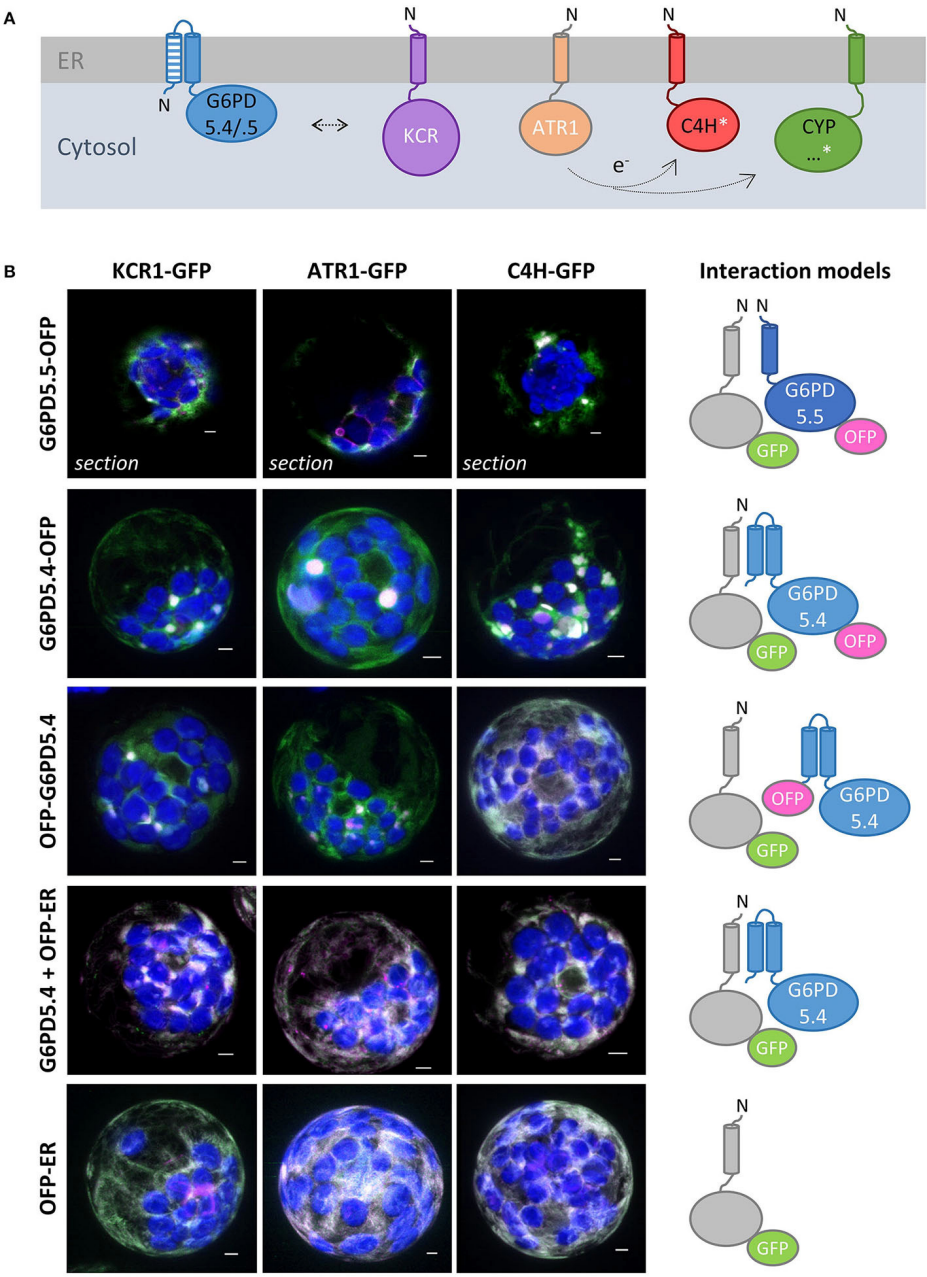
NADPH-dependent enzymes co-localize with membrane-bound G6PD5 in ER subdomains. **(A)** Scheme of membrane-bound enzymes that consume NADPH at the cytosolic face of the ER, either directly (white) or indirectly (white asterisk): KCR1, ketoacyl-CoA reductase (part of the fatty acid elongase complex); ATR1, NADPH-dependent cytochrome P450 oxidoreductase (reduces cytochrome P450 enzymes, CYP) like C4H/CYP73A5 (cinnamate 4-hydroxylase, involved in the biosynthesis of phenylpropanes) and found upon GFP-G6PD5.4 pull-down (for ratiometric *ro*GFP analyses, confirming that the catalytic domains face the cytosol, refer to Supplementary Figure S8). **(B)** Co-expression of the GFP fusion proteins with either G6PD5.5-OFP, G6PD5.4-OFP, or N-terminally tagged OFP-G6PD5.4, and with the soluble luminal ER marker (OFP-ER) with label-free G6PD5.4, compared to OFP-ER alone (bottom). If not stated otherwise, the images show maximal projections of about 30 optical sections as a merger of all channels, (for single channel images, refer to Supplementary Figure S9). GFP in green, OFP in magenta, and chlorophyll auto-fluorescence in blue. White signals indicate co-localization of GFP and OFP (or very close signals < 200 nm). Scale bars, 3 μm.

Since among the pulled-down proteins, two cytochrome P450 enzymes (C4H/CYP73A5 and CYP71B6) were enriched as well (Figure 8D), we wondered whether the tendency of the membrane-bound G6PD5 proteoforms to cluster with the other two OPPP enzymes in ER subdomains may extend to enzymes of anabolic pathways that operate at the cytosolic face of the ER. Thus, representative candidates that either depend directly or indirectly on NADPH provision (Figure 9A) were cloned as C-terminal *ro*GFP fusion, namely, KCR1 (ketoacyl-CoA reductase, involved in the elongation of fatty acids), ATR1 (NADPH:cytochrome-P450 oxidoreductase), and C4H/CYP73A5 (cinnamate 4-hydroxylase, obtained by GFP-G6PD5.4 pull-down) as indirectly NADPH-dependent cytochrome P450 enzyme (*via* ATR). Expression in Arabidopsis wild-type protoplasts confirmed that the catalytic domains face the cytosol (Supplementary Figure S8). When expressed on their own – the candidate *ro*GFP fusions did not show a strong tendency to cluster at the ER and displayed even ER labeling when co-expressed with a soluble ER marker (OFP-ER; Figure 9B, bottom), which contrasts with the results obtained for the membrane-bound G6PD5 proteoforms (Figure 4A). When co-expressed with different OFP fusions of the membrane-bound G6PD5.4 proteoform, KCR1-, ATR1-, and C4H-*ro*GFP accumulated in the same ER subdomains, preferentially with G6PD5.4-OFP (Figure 9B), and still when using label-free G6PD5.4 co-expressed with OFP-ER (in the Nxn site). Yet, G6PD5.5-OFP showed only limited overlap with the candidate *ro*GFP fusions (Figure 9B, top), preferentially labeling distinct ER subdomains, especially when co-expressed with ATR1-*ro*GFP (for single channel images, refer to Supplementary Figure S9). Hence, alternative splicing of *G6PD5* targets the proteoforms to different ER subdomains, which is also reflected by selective co-localization with key enzymes of membrane-bound enzyme assemblies at the ER.

**Figure 10:**
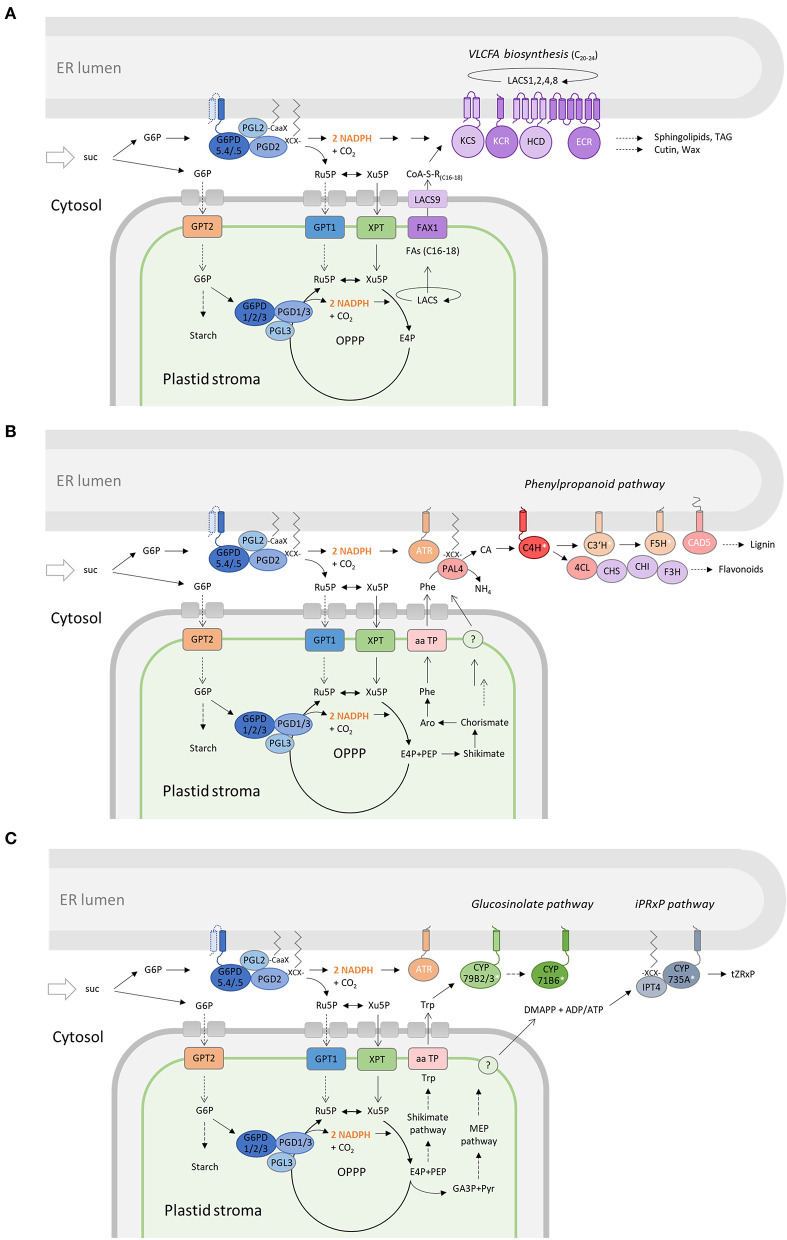
Membrane-bound OPPP metabolons facilitate NADPH provision at the cytosolic face of the ER. Cooperation of the OPPP metabolon at the ER (with the complete OPPP cycle in plastids) to support membrane-bound pathways that consume NADPH in plant cells. **(A)** KCR1 (and ECR) are NADPH-dependent members (white) of the long-chain acyl-CoA synthase complex at the ER (LACS1,2,4,8). Chain elongation leads to very long-chain fatty acids (VLCFAs) as precursors of sphingolipids, triacylglycerols (TAG), cutins, and waxes. **(B)** In the phenylpropanoid pathway (starting from plastid-exported phenylalanine, Phe), CAD5 is directly NADPH-dependent, whereas C4H, C3H, and F5H are cytochrome P450 enzymes (white asterisks) that depend on NADPH:cytochrome P450 oxidoreductase (ATR) for reduction in the synthesis of lignin and flavonoids. **(C)** Several enzymes of the glucosinolate pathway (CYP79B2/3, CYP71B6) lead from plastid-exported tryptophan (Trp) to defense compounds, or in roots *via* the iPRxP pathway to active cytokinins (*trans*-Zeatin) *via* IPT4 (palmitoylation weakly predicted) and CYP735A that indirectly depends on NADPH (*via* ATR). DMAPP, dimethylallyl-diphosphate; E4P, erythrose-4-phosphate; suc, sucrose; CA, cinnamic acid; TAG, triacylglycerol; tZRxP, trans-Zeatin-ribose phosphates.

In any case, OPPP metabolon formation at the cytosolic face of the ER should facilitate local NADPH provision under conditions of high sugar availability, especially in sink situations that are characterized by sucrose import or retention during stress (Figure 10).

## Discussion

Metabolon formation at the endoplasmic reticulum of plant cells has lately received attention, since membrane-steroid-binding proteins MSBP1 and MSBP2 were reported to form a scaffold for a subset of monolignol cytochrome-P450 enzymes that synthesize lignin precursors at the cytosolic face of the ER (Gou et al., 2018). Predicted membrane topology for the two MSBP proteins is “N-out” – with the C-terminal domains facing the cytosol – and not the ER lumen, as suggested by the scheme in Wang and Zhao (2018). We tested split YFP fusions with the same membrane topology in our protoplast system, but already the empty vector controls of the split YFP combinations gave strong cytosolic signals (Supplementary Figure S10, bottom). Nevertheless, G6PD5.4-YFP^N^ recruited empty ^C^YFP to ER subdomains. Thus, although informative, BiFC studies at the cytosolic face of the ER seem problematic, as previously shown by the analyses of proteins involved in ER-plasma membrane tethering in plant cells (Tao et al., 2019).

Overexpression of polytopic membrane proteins often results in clustering at the ER, especially when enforced by dimerization *via* split YFP reconstitution, which we exploited for GPT1 in Baune et al. (2020). As shown here, also proteins with only one or two membrane-spanning domains may transform the ER from a network of branching tubules to a variable number of stacked or folded membrane arrays – besides the known ER-shaping protein classes Reticulon, Lunapark, and Atlastin (reviewed in Kriechbaumer and Brandizzi, 2020). Other prominent examples are the isoforms of HMG-CoA reductase (HMGR) with two N-terminal transmembrane domains (Leivar et al., 2005; Ferrero et al., 2015; Grados-Torrez et al., 2021) and splice variant YUC4.2 with C-terminal tail anchor that is mainly expressed in Arabidopsis flowers (Kriechbaumer et al., 2012). Initially analyzed in plant cells (termed “membrane zippering”; Mullen et al., 2001), and later coined OSER (for “organized smooth endoplasmic reticulum”) based on the studies in yeast and animal cells (Snapp et al., 2003), the formation of ER subdomains is a dynamic event, mainly governed by the principles of self-organization (Borgese et al., 2006). While recognition of the phenomenon dates back to EM studies in the 1980s, the formation of OSER structures applies to all eukaryotes (reviewed in Sandor et al., 2021). One open question still is whether specific ER proteins are needed for creating/maintaining (native) OSER structures.

As shown by Snapp et al. (2003), GFP attached to a proteinaceous membrane anchor (replacing the catalytic domain) may diffuse freely in and out of OSER structures. But not all proteins at the cytosolic face of the ER induce these structures. For enzymes of the phenylpropanoid pathway, weak interactions had to be documented by FRET analyses (Bassard et al., 2012), since co-expression of L-phenylalanine ammonia-lyase (PAL) and cinnamate 4-hydroxylase (C4H/CYP73A5, for metabolic channeling; Achnine et al., 2004) did not show a strong tendency to form substructures at the ER, and co-expression with MSBP proteins neither (Gou et al., 2018). Even CPR/POR enzymes of poplar, required for the reduction of cytochrome P450 enzymes (like C4H/CYP73A5), did not accumulate in ER subdomains (Ro et al., 2002). More recently, interaction among synthetic anchors of CPR/POR enzymes (called ATR in Arabidopsis) was investigated *in vitro* by single-molecule tracking in the lipid phase (Laursen et al., 2021). These authors found that the membrane environment can enhance protein concentration, which led them to conclude that high conservation among the transmembrane sequences of ATR/CPR/POR in different land plants follows an amino acid consensus, which differed from the one suggested for HMGR enzymes with low sequence similarity among eukaryotic kingdoms (Ferrero et al., 2015). When comparing the membrane region linked to the catalytic G6PD domains, G6PD5.4 (PISLNFNFTGLFCILVRVLk) and G6PD5.5 (eNFVATVAGLFCILVRVLk) show only partial conservation (identical amino acids underlined). Actually, when expressed on their own, the two proteoforms accumulated in different ER subdomains, which partially may be due to the presence of a second membrane domain in the N-terminus of G6PD5.4. Of note, ATR1 did not cluster when co-expressed with a luminal ER marker in protoplasts, and although displaying only a single transmembrane domain - like G6PD5.5, did not co-localize with this proteoform in ER substructures, but with G6PD5.4. When only the N-terminal G6PD5.4 domain was used, there was still a tendency for the reporter fusions to accumulate in focal ER sites. Hence, similar to HMGR with two N-terminal transmembrane regions and multimerization among the catalytic domains (Friesen and Rodwell, 2004; Leivar et al., 2005; Grados-Torrez et al., 2021), the membrane-bound G6PD5 proteoforms tend to cluster at the ER, likely promoted by dimerization – and when this occurs between proteins in opposing membranes, OSER structures are formed (Sandor et al., 2021). Thus, the spatial organization of the membrane-bound G6PD5 proteoforms at the ER seems to be governed by features of both, the transmembrane regions and the globular catalytic domains.

Overexpression of active G6PD5 should lead to accumulation of 6-phosphogluconolactone (6PL), and – if not channeled instantly by a PGL (Lansing et al., 2020), the δ-6PL form will spontaneously convert to the χ-form, a dead-end of metabolism (Miclet et al., 2001). Co-localization of the N-terminally extended G6PD5 proteoforms with C-terminally prenylated PGL2 in ER subdomains should diminish 6PL release, and as indicated by our pull-down analyses, physical interaction with palmitoylated PGD2 completes the membrane-bound OPPP metabolon. Reporter-G6PD5.4 fusions tended to form clusters, irrespective of an additional presence of PGL2 or PGD2. In fact, co-expression of GFP-G6PD5.4 with OFP-G6PD5.4 increased co-localization in the same ER subdomains, which supports that the induced OSER structures mainly result from the interaction of the catalytic domains. Since cytosolic G6PD5.1 or PGL2 with mutated prenylation motif were recruited less – or not at all (Figures 3C,D, 6), interaction at the ER seems to occur mainly between membrane-bound proteins. Notably, S-acylation of cysteine residues is known to promote membrane association and protein–protein interaction (reviewed in Li and Qi, 2017). This is in line with our GFP-based pull-down analyses, which showed that both PGL2 and PGD2 co-purified with GFP-G6PD5.4 (Figure 8). Failure of PGL2 detection on immunoblots seems to be due to post-translational modification *in planta*, most likely C-terminal prenylation.

The concentration of G6PD5.5 and G6PD5.4 in distinct ER subdomains might well serve regulatory functions. Alternative splicing is known to be triggered mainly by stress in plants (Mastrangelo et al., 2012; Kornblihtt et al., 2013; Martín et al., 2021), and compared to *G6PD5.5*, the *G6PD5.4* splice form was more abundant under a physiological sink situation (1% sucrose, Figure 1B). Moreover, as shown by our transient transfection of naïve protoplasts, OSER structures are formed upon sudden expression waves of the membrane-bound G6PD5 proteoforms. If PGL2 should not immediately be recruited (or upregulated due to linkage: *G6PD5* At3g27300g, *PGL2* At3g49360, and *PGD2* At3g02360), this may lead to temporarily elevated δ-6PL levels that spontaneously convert to the χ-form. Interestingly, a function for this dead-end metabolite has recently been uncovered in human lung cancer cells (Gao et al., 2019). Accumulation of the χ-form enhanced inhibitory phosphorylation of a protein phosphatase (PP2A), which interfered with AMPK signaling. The AMPK homolog of plants is sucrose non-fermenting-1-related protein kinase-1 (SnRK1), a well-known energy-sensing/signaling complex for the coordination of metabolic pathways during stress adaptation and growth (reviewed in Rodriguez et al., 2019). However, whether the above described regulation principle extends to plant cells remains to be investigated.

To this end, our studies show that the N-terminally extended G6PD5 proteoforms recruit an NADPH-producing OPPP metabolon to the cytosolic face of the ER. G6PD5.4 and G6PD5.5 accumulated in discrete ER subdomains, where they may cooperate with different metabolic pathways – possibly *via* the distantly related and differently regulated ATR1 (constitutive) and ATR2 (stress-induced) isoforms (Urban et al., 1997; Mizutani and Ohta, 1998). Both membrane-bound G6PD5 variants were active on their own (by forming homodimers) and induced OSER structures. Together with the recruitment of the lipid-linked PGL2 and PGD2 isoforms, they form the basis for sugar-derived NADPH provision to plant pathways that operate as metabolons at the cytosolic face of the ER (e.g., fatty acid elongation) or loose enzyme assemblies (e.g., among the phenylpropanoid pathway). In fact, the biosynthesis of anthocyanins and flavonoids is induced by sucrose (Solfanelli et al., 2006). This may be linked to stress (such as pathogen attack, reviewed in Moghaddam and Van den Ende, 2012), but does not include a membrane-bound hexokinase, since AS variant HXK2.1-*ro*GFP co-localized with mitochondria (Supplementary Figure S11), such as HXK1 (Kim et al., 2006) among the highly active HXK isoforms (Karve et al., 2008).

Several enzymes of the lipid-elongase complex at the ER that synthesize very long-chain fatty acids (VLCFAs) from plastid exported precursors (Figure 10A) depend on NADPH provision (Haslam and Kunst, 2013), and also a fatty acid epoxidase (CYP77B1) and a fatty acid hydrolase (CYP703A2/DEX2) are among the indirectly NADPH-dependent cytochrome P450 (CYP) enzymes. CYPs are reduced by NADPH-hemoprotein oxidoreductases (ATR in Arabidopsis) and include those for the biosynthesis of several important defense compounds (e.g., Camalexin; Böttcher et al., 2014), such as CYP79B2 or 3 and CYP71B6 (Figure 10C). The latter was detected among the GFP-G6PD5.4 pulled-down candidates from mesophyll protoplasts (Figure 8D). Yet, also biosynthesis, degradation, and/or inactivation of phytohormones may involve CYP enzymes, e.g., the formation of *trans*-Zeatin *via* two CYP735A isoforms (Takei et al., 2004) that are mainly expressed in roots. On the other hand, CYP707A enzymes are abscisic acid 8-hydroxylases (Okamoto et al., 2006), CYP72A isoforms gibberellin 13-hydroxylases (He et al., 2019), whereas CYP72C1/SOB7 is involved in brassinosteroid homeostasis (Nakamura et al., 2005; Takahashi et al., 2005; Turk et al., 2005), and CYP711A1/MAX1 in strigolactone biosynthesis/signaling (Booker et al., 2005).

In summary, especially during high metabolic flux, the irreversible OPPP reactions need to operate as metabolon, which was originally proposed for the two dehydrogenases in plastids (Debnam et al., 1997) and later shown to include dually targeted PGL3 (Xiong et al., 2009) – also in peroxisomes (Lansing et al., 2020). Albeit the cytosolic part of the OPPP is not essential in Arabidopsis, since *g6pd5 g6pd6* double mutants are viable (Wakao et al., 2008; this study), the membrane-bound G6PD5 proteoforms generated by AS in wild-type plants should be beneficial under certain conditions – such as photoautotrophic growth (G6PD5.5) vs. stress-induced physiological sink states (G6PD5.4) that are characterized by sucrose backup (Scharte et al., 2009). Furthermore, metabolon formation may additionally be influenced by post-translational modifications. In fact, phosphorylation of a conserved threonine was shown to activate cytosolic G6PD6 and also G6PD5 *in vitro* (Dal Santo et al., 2012). A putative role for OPPP metabolon formation should be studied in comparison with other compartments – in the context of cooperation (i.e. ER membrane with plastids, Figure 10) – and also with potential other metabolic pathways. For example, the early steps of isoprenoid biosynthesis (leading to specialized, secondary metabolites that participate in interactions with the environment) are distributed across several plant cell compartments (Simkin et al., 2011; reviewed in Pulido et al., 2012). Pace-making HMGR depends on NADPH provision at the cytosolic face of the ER, but follow-up enzymes operate in the cytosol and/or peroxisomes, before different products (sterols, dolichols, etc.) are finalized at the hydrophobic interface of the ER membrane. Links to energy provision at the different cellular locations are therefore fascinating aspects for future studies.

## Data availability statement

The original contributions presented in the study are included in the article/Supplementary material, further inquiries can be directed to the corresponding author.

## Author contributions

LL, LD, HL, and AvS jointly developed the project. LL, LD, and KF performed the experiments. AvS wrote the paper with editorial contributions of LL, LD, HL, JE, and IF. All authors analyzed data and approved the final version of the manuscript.

## Funding

This work was supported financially by the DFG (Deutsche Forschungsgemeinschaft), by grant SCHA 541/12-3 to AvS and grant INST 211/744-1 FUGG to IF for LC-MS/MS analyses.

## Conflict of interest

The authors declare that the research was conducted in the absence of any commercial or financial relationships that could be construed as a potential conflict of interest.

## Publisher's note

All claims expressed in this article are solely those of the authors and do not necessarily represent those of their affiliated organizations, or those of the publisher, the editors and the reviewers. Any product that may be evaluated in this article, or claim that may be made by its manufacturer, is not guaranteed or endorsed by the publisher.

## References

[B1] Achnine L. Blancaflor E. B. Rasmussen S. Dixon R. A. (2004). Colocalization of L-phenylalanine ammonia-lyase and cinnamate 4-hydroxylase for metabolic channeling in phenylpropanoid biosynthesis. Plant Cell 16, 3098–3109. 10.1105/tpc.104.024406 15472080 PMC527201

[B2] Bassard J.-E. Richert L. Geerinck J. Renault H. Duval F. Ullmann P.. (2012). Protein-protein and protein-membrane associations in the lignin pathway. Plant Cell 24, 4465–4482. 10.1105/tpc.112.102566 23175744 PMC3531846

[B3] Baune M.-C. Lansing H. Fischer K. Meyer T. Charton L. Linka N.. (2020). The *Arabidopsis* plastidial glucose-6-phosphate transporter GPT1 is dually targeted to peroxisomes *via* the endoplasmic reticulum. Plant Cell 32, 1703–1726. 10.1105/tpc.19.00959 32111666 PMC7203913

[B4] Booker J. Sieberer T. Wright W. Williamson L. Willett B. Stirnberg P.. (2005). MAX1 encodes a cytochrome P450 family member that acts downstream of MAX3/4 to produce a carotenoid-derived branch-inhibiting hormone. Dev. Cell 8, 443–449. 10.1016/j.devcel.2005.01.009 15737939

[B5] Borgese N. Francolini M. Snapp E. (2006). Endoplasmic reticulum architecture: structures in flux. Curr. Opin. Cell Biol. 18, 358–364. 10.1016/j.ceb.2006.06.008 16806883 PMC4264046

[B6] Böttcher C. Chapman A. Fellermeier F. Choudhary M. Scheel D. Glawischnig E.. (2014). The biosynthetic pathway of indole-3-carbaldehyde and indole-3-carboxylic acid derivatives in *Arabidopsis*. Plant Physiol. 165, 841–853. 10.1104/pp.114.235630 24728709 PMC4044862

[B7] Chaudhary S. Khokhar W. Jabre I. Reddy A. S. N. Byrne L. J. Wilson C. M.. (2019). Alternative splicing and protein diversity: plants versus animals. Front. Plant Sci. 10:708. 10.3389/fpls.2019.00708 31244866 PMC6581706

[B8] Dal Santo S. Stampfl H. Krasensky J. Kempa S. Gibon Y. Petutschnig E.. (2012). Stress-induced GSK3 regulates the redox stress response by phosphorylating glucose-6-phosphate dehydrogenase in *Arabidopsis*. Plant Cell 24, 3380–3392. 10.1105/tpc.112.101279 22885737 PMC3462638

[B9] Debnam P. M. Shearer G. Blackwood L. Kohl D. H. (1997). Evidence for channeling of intermediates in the oxidative pentose phosphate pathway by soybean and pea nodule extracts, yeast extracts, and purified yeast enzymes. Eur. J. Biochem. 246, 283–290. 10.1111/j.1432-1033.1997.00283.x 9208916

[B10] Dietz K.-J. (2014). Redox regulation of transcription factors in plant stress acclimation and development. Antioxid. Redox Signal. 21, 1356–1372. 10.1089/ars.2013.5672 24182193

[B11] Duy D. Soll J. Philippar K. (2007). Solute channels of the outer membrane: from bacteria to chloroplasts. Biol. Chem. 388, 879–889. 10.1515/BC.2007.120 17696771

[B12] Ferrero S. Grados-Torrez R. E. Leivar P. Antolín-Llovera M. Lǿpez-Iglesias C. Cortadellas N.. (2015). Proliferation and morphogenesis of the endoplasmic reticulum driven by the membrane domain of 3-hydroxy-3-methylglutaryl coenzyme a reductase in plant cells. Plant Physiol. 168, 899–914. 10.1104/pp.15.00597 26015445 PMC4741317

[B13] Friesen J. A. Rodwell V. W. (2004). The 3-hydroxy-3-methylglutaryl coenzyme-A (HMG-CoA) reductases. Genom. Biol. 5, 248. 10.1186/gb-2004-5-11-248 15535874 PMC545772

[B14] Gao X. Zhao L. Liu S. Li Y. Xia S. Chen D.. (2019). γ-6-Phosphogluconolactone, a byproduct of the oxidative pentose phosphate pathway, contributes to AMPK activation through inhibition of PP2A. Mol. Cell 76, 857–871.e9. 10.1016/j.molcel.2019.09.007 31586547 PMC6925637

[B15] Gou M. Ran X. Martin D. W. Liu C.-J. (2018). The scaffold proteins of lignin biosynthetic cytochrome P450 enzymes. Nat. Plants 4, 299–310. 10.1038/s41477-018-0142-9 29725099

[B16] Graciet E. Gans P. Wedel N. Lebreton S. Camadro J. M. Gontero B.. (2003). The small protein CP12: a protein linker for supramolecular complex assembly. Biochemistry 42, 8163–8170. 10.1021/bi034474x 12846565

[B17] Grados-Torrez R. E. López-Iglesias C. Ferrer J. C. Campos N. (2021). Loose morphology and high dynamism of OSER structures induced by the membrane domain of HMG-CoA reductase. Int. J. Mol. Sci. 22, 9132. 10.3390/ijms22179132 34502042 PMC8430881

[B18] Haslam T. M. Kunst L. (2013). Extending the story of very-long-chain fatty acid elongation. Plant Sci. 210, 93–107. 10.1016/j.plantsci.2013.05.008 23849117

[B19] Hauschild R. von Schaewen A. (2003). Differential regulation of glucose-6-phosphate dehydrogenase isoenzyme activities in potato. Plant Physiol. 133, 47–62. 10.1104/pp.103.025676 12970474 PMC196576

[B20] He J. Chen Q. Xin P. Yuan J. Ma Y. Wang X.. (2019). CYP72A enzymes catalyse 13-hydrolyzation of gibberellins. Nat. Plants 5, 1057–1065. 10.1038/s41477-019-0511-z 31527846 PMC7194175

[B21] Hemsley P. A. Weimar T. Lilley K. S. Dupree P. Grierson C. S. (2013). A proteomic approach identifies many novel palmitoylated proteins in *Arabidopsis*. New Phytol. 197, 805–814. 10.1111/nph.12077 23252521

[B22] Hölscher C. Lutterbey M.-C. Lansing H. Meyer T. Fischer K. von Schaewen A.. (2016). Defects in peroxisomal 6-phosphogluconate dehydrogenase isoform PGD2 prevent gametophytic interaction in *Arabidopsis thaliana*. Plant Physiol. 171, 192–205. 10.1104/pp.15.01301 26941195 PMC4854672

[B23] Hölscher C. Meyer T. von Schaewen A. (2014). Dual-targeting of *Arabidopsis* 6-phosphogluconolactonase 3 (PGL3) to chloroplasts and peroxisomes involves interaction with Trx m2 in the cytosol. Mol. Plant 7, 252–255. 10.1093/mp/sst126 24008768

[B24] Hu J. Baker A. Bartel B. Linka N. Mullen R. T. Reumann S.. (2012). Plant peroxisomes: biogenesis and function. Plant Cell 24, 2279–2303. 10.1105/tpc.112.096586 22669882 PMC3406917

[B25] Hughes C. S. Moggridge S. Müller T. (2019). Single-pot, solid-phase-enhanced sample preparation for proteomics experiments. Nat. Protoc. 14, 68–85. 10.1038/s41596-018-0082-x 30464214

[B26] Jørgensen K. Vinther Rasmussen A. Morant M. Holm Nielsen A. Bjarnholt N. Zagrobelny M.. (2005). Metabolon formation and metabolic channeling in the biosynthesis of plant natural products. Curr. Opin. Plant Biol. 8, 280–291. 10.1016/j.pbi.2005.03.014 15860425

[B27] Karve A. Rauh B. L. Xia X. Kandasamy M. Meagher R. B. Sheen J.. (2008). Expression and evolutionary features of the hexokinase gene family in *Arabidopsis*. Planta 228, 411–425. 10.1007/s00425-008-0746-9 18481082 PMC2953952

[B28] Kim M. Lim J. H. Ahn C. S. Park K. Kim G. T. Kim W. T.. (2006). Mitochondria-associated hexokinases play a role in the control of programmed cell death in Nicotiana benthamiana. Plant Cell 18, 2341–2355. 10.1105/tpc.106.041509 16920781 PMC1560927

[B29] Kornblihtt A. R. Schor I. E. All,ó M. Dujardin G. Petrillo E. Muñoz M. J.. (2013). Alternative splicing: a pivotal step between eukaryotic transcription and translation. Nat. Rev. Mol. Cell Biol. 14, 153–165. 10.1038/nrm3525 23385723

[B30] Kriechbaumer V. Brandizzi F. (2020). The plant endoplasmic reticulum: an organized chaos of tubules and sheets with multiple functions. J. Microscopy 280, 122–133. 10.1111/jmi.12909 32426862 PMC10895883

[B31] Kriechbaumer V. Wang P. Hawes C. Abell B. M. (2012). Alternative splicing of the auxin biosynthesis gene YUCCA4 determines its subcellular compartmentation. Plant J. 70, 292–302. 10.1111/j.1365-313X.2011.04866.x 22233288

[B32] Kruger N. J. von Schaewen A. (2003). The oxidative pentose phosphate pathway: structure and organization. Curr. Opin. Plant Biol. 6, 236–246. 10.1016/S1369-5266(03)00039-6 12753973

[B33] KumarM.CarrP.TurnerS. (2020). (2020). An atlas of *Arabidopsis* protein S-Acylation reveals its widespread role in plant cell organisation of and function. bioRxiv. 10.1101/2020.05.12.09041535681017

[B34] Lansing H. Doering L. Fischer K. Baune M.-C. von Schaewen A. (2020). Analysis of potential redundancy among *Arabidopsis* 6-phosphogluconolactonase isoforms in peroxisomes. J. Exp. Bot. 71, 823–836. 10.1093/jxb/erz473 31641750

[B35] Laursen T. Lam H. Y. M. Sørensen K. K. Tian P. Hansen C. C. Groves J. T.. (2021). (2021). Membrane anchoring facilitates colocalization of enzymes in plant cytochrome P450 redox systems. Commun. Biol. 4, 1057. 10.1038/s42003-021-02604-1 34504298 PMC8429664

[B36] Leivar P. González V. M. Castel S. Trelease R. N. López-Iglesias C. Arró M.. (2005). Subcellular localization of *Arabidopsis* 3-hydroxy-3-methylglutaryl-coenzyme A reductase. Plant Physiol. 137, 57–69. 10.1104/pp.104.050245 15618432 PMC548838

[B37] Li Y. Qi B. (2017). Progress toward understanding protein s-acylation: Prospective in plants. Front. Plant Sci. 8, 346. 10.3389/fpls.2017.00346 28392791 PMC5364179

[B38] Marquez Y. Brown J. W. S. Simpson C. Barta A. Kalyna (2012). Transcriptome survey reveals increased complexity of the alternative splicing landscape in *Arabidopsis*. Genome Res. 22, 1184–1195. 10.1101/gr.134106.111 22391557 PMC3371709

[B39] Martín G. Márquez Y. Mantica F. Duque P. Irimia M. (2021). Alternative splicing landscapes in *Arabidopsis thaliana* across tissues and stress conditions highlight major functional differences with animals. Genome Biol. 22, 35. 10.1186/s13059-020-02258-y 33446251 PMC7807721

[B40] Mastrangelo A. M. Marone D. Laid,ò G. De Leonardis A. M. De Vita P. (2012). Alternative splicing: enhancing ability to cope with stress *via* transcriptome plasticity. Plant Sci. 185–186, 40–49. 10.1016/j.plantsci.2011.09.006 22325865

[B41] Meyer T. Hölscher C. Schwöppe C. von Schaewen A. (2011). Alternative targeting of *Arabidopsis* plastidic glucose-6-phosphate dehydrogenase G6PD1 involves cysteine-dependent interaction with G6PD4 in the cytosol. Plant J. 66, 745–758. 10.1111/j.1365-313X.2011.04535.x 21309870

[B42] Miclet E. Stoven V. Michels P. A. M. Opperdoes F. R. Lallemand J. -Y. Duffieux F. (2001). NMR spectroscopic analysis of the first two steps of the pentose-phosphate pathway elucidates the role of 6-phosphogluconolactonase. J. Biol. Chem. 276, 34840–34846. 10.1074/jbc.M105174200 11457850

[B43] Mittler R. (2017). ROS are good. Trends Plant Sci. 22, 1–19. 10.1016/j.tplants.2016.08.002 27666517

[B44] Mizutani M. Ohta D. (1998). Two isoforms of NADPH:cytochrome P450 reductase in *Arabidopsis thaliana*. Gene structure, heterologous expression in insect cells, and differential regulation. Plant Physiol. 116, 357–367. 10.1104/pp.116.1.357 9449848 PMC35176

[B45] Moghaddam M. R. B. Van den Ende W. (2012). Sugars and plant innate immunity. J. Exp. Botany 63, 3989–3998. 10.1093/jxb/ers129 22553288

[B46] Mullen R. T. Lisenbee C. S. Flynn R. C. Trelease R. N. (2001). Stable and transient expression of chimeric peroxisomal membrane proteins induces an independent “zippering” of peroxisomes and an endoplasmic reticulum subdomain. Planta 213, 849–863. 10.1007/s004250100579 11722121

[B47] Nakamura M. Satoh T. Tanaka S. Mochizuki N. Yokota T. Nagatani A.. (2005). Activation of the cytochrome P450 gene, CYP72C1, reduces the levels of active brassinosteroids *in vivo*. J. Exp. Bot. 56, 833–840. 10.1093/jxb/eri073 15689343

[B48] Née G. Zaffagnini M. Trost P. Issakidis-Bourguet E. (2009). Redox regulation of chloroplastic glucose-6-phosphate dehydrogenase: a new role for f-type thioredoxin. FEBS Lett. 583, 2827–2832. 10.1016/j.febslet.2009.07.035 19631646

[B49] Okamoto M. Kuwahara A. Seo M. Kushiro T. Asami T. Hirai N.. (2006). CYP707A1 and CYP707A2, which encode abscisic acid 8-hydroxylases, are indispensable for proper control of seed dormancy and germination in *Arabidopsis*. Plant Physiol. 141, 97–107. 10.1104/pp.106.079475 16543410 PMC1459320

[B50] Okuda S. Watanabe Y. Moriya Y. Kawano S. Yamamoto T. Matsumoto M.. (2017). jPOSTrepo: an international standard data repository for proteomes. Nucl. Acids Res. 45, D1107–D1111. 10.1093/nar/gkw1080 27899654 PMC5210561

[B51] Pugin A. Frachisse J. M. Tavernier E. Bligny R. Gout E. Douce R.. (1997). Early events induced by the elicitor Cryptogein in tobacco cells: involvement of a plasma membrane NADPH oxidase and activation of glycolysis and the pentose phosphate pathway. Plant Cell 9, 2077–2091. 10.2307/3870566 12237354 PMC157059

[B52] Pulido P. Perello C. Rodriguez-Concepcion M. (2012). New insights into plant isoprenoid metabolism. Mol. Plant 5, 964–967. 10.1093/mp/sss088 22972017

[B53] Reddy A. (2007). Alternative splicing of pre-messenger RNAs in plants in the genomic era. Annu. Rev. Plant Biol. 58, 267–361. 10.1146/annurev.arplant.58.032806.103754 17222076

[B54] Ro D. K. Ehlting J. Douglas C. J. (2002). Cloning, functional expression, and subcellular localization of multiple NADPH-cytochrome P450 reductases from hybrid poplar. Plant Physiol. 130, 1837–1851. 10.1104/pp.008011 12481067 PMC166695

[B55] Rodriguez M. Parola R. Andreola S. Pereyra C. Martínez-Noël G. (2019). TOR and SnRK1 signaling pathways in plant response to abiotic stresses: do they always act according to the “yin-yang” model? Plant Sci. 288, 110220. 10.1016/j.plantsci.2019.110220 31521220

[B56] Sandor A. Fricker M. D. Kriechbaumer V. Sweetlove L. J. (2021). IntEResting structures: formation and applications of organized smooth endoplasmic reticulum in plant cells. Plant Physiol. 185, 550–561. 10.1104/pp.20.00719 33822222 PMC8892044

[B57] Scharte J. Schön H. Tjaden Z. Weis E. von Schaewen A. (2009). Isoenzyme replacement of glucose-6-phosphate dehydrogenase in the cytosol improves stress tolerance in plants. Proc. Natl. Acad. Sci. USA 106, 8061–8066. 10.1073/pnas.0812902106 19416911 PMC2683143

[B58] Scheibe R. (1990). Light/dark modulation: regulation of chloroplast metabolism in a new light. Bot. Acta 103, 327–334. 10.1111/j.1438-8677.1990.tb00170.x

[B59] Schnarrenberger C. Flechner A. Martin W. (1995). Enzymatic evidence for a complete oxidative pentose phosphate pathway in chloroplasts and an incomplete pathway in the cytosol of spinach leaves. Plant Physiol. 108, 609–614. 10.1104/pp.108.2.609 12228497 PMC157380

[B60] Simkin A. J. Guirimand G. Papon N. Courdavault V. Thabet I. Ginis O.. (2011). Peroxisomal localisation of the final steps of the mevalonic acid pathway in planta. Planta 234, 903–914. 10.1007/s00425-011-1444-6 21655959

[B61] Snapp E. L. Hegde R. S. Francolini M. Lombardo F. Colombo S. Pedrazzini E.. (2003). Formation of stacked ER cisternae by low affinity protein interactions. J. Cell Biol. 163, 257–269. 10.1083/jcb.200306020 14581454 PMC2173526

[B62] Solfanelli C. Poggi A. Loreti E. Alpi A. Perata P. (2006). Sucrose-specific induction of the anthocyanin biosynthetic pathway in *Arabidopsis*. Plant Physiol. 140, 637–646. 10.1104/pp.105.072579 16384906 PMC1361330

[B63] Stincone A. Prigione A. Cramer T. Wamelink M. M. C. Campbell K. Cheung E.. (2015). The return of metabolism: biochemistry and physiology of the pentose phosphate pathway. Biol. Rev. 90, 927–963. 10.1111/brv.12140 25243985 PMC4470864

[B64] Takahashi N. Nakazawa M. Shibata K. Yokota T. Ishikawa A. Suzuki K.. (2005). shk1-D, a dwarf *Arabidopsis* mutant caused by activation of the CYP72C1 gene, has altered brassinosteroid levels. Plant J. 42, 13–22. 10.1111/j.1365-313X.2005.02357.x 15773850

[B65] Takei K. Yamaya T. Sakakibara H. (2004). *Arabidopsis* CYP735A1 and CYP735A2 encode cytokinin hydroxylases that catalyze the biosynthesis of trans-Zeatin. J. Biol. Chem. 279, 41866–41872. 10.1074/jbc.M406337200 15280363

[B66] Tao K. Waletich J. R. Arredondo F. Tyler B. M. (2019). Manipulating endoplasmic reticulum-plasma membrane tethering in plants through fluorescent protein complementation. Front. Plant Sci. 10, 635. 10.3389/fpls.2019.00635 31191568 PMC6547045

[B67] Turk E. M. Fujioka S. Seto H. Shimada Y. Takatsuto S. Yoshida S.. (2005). BAS1 and SOB7 act redundantly to modulate *Arabidopsis* photomorphogenesis *via* unique brassinosteroid inactivation mechanisms. Plant J. 42, 23–34. 10.1111/j.1365-313X.2005.02358.x 15773851

[B68] Tyanova S. Temu T. Cox J. (2016). The MaxQuant computational platform for mass spectrometry-based shotgun proteomics. Nat. Protoc. 11, 2301–2319. 10.1038/nprot.2016.136 27809316

[B69] Urban P. Mignotte C. Kazmaier M. Delorme F. Pompon D. (1997). Cloning, yeast expression, and characterization of the coupling of two distantly related *Arabidopsis thaliana* NADPH-cytochrome P450 reductases with P450 CYP73A5. J. Biol. Chem. 272, 19176–19186. 10.1074/jbc.272.31.19176 9235908

[B70] Van Breusegem F. Vranová E. Dat J. F. Inzé D. (2001). The role of active oxygen species in plant signal transduction. Plant Sci. 161, 405–414. 10.1016/S0168-9452(01)00452-6

[B71] Wakao S. Andre C. Benning C. (2008). Functional analyses of cytosolic glucose-6-phosphate dehydrogenases and their contribution to seed oil accumulation in *Arabidopsis*. Plant Physiol. 146, 277–288. 10.1104/pp.107.108423 17993547 PMC2230552

[B72] Wakao S. Benning C. (2005). Genome-wide analysis of glucose-6-phosphate dehydrogenases in *Arabidopsis*. Plant J. 41, 243–256. 10.1111/j.1365-313X.2004.02293.x 15634201

[B73] Wang B. Zhao Q. (2018). Plant secondary metabolism - membrane-bound metabolons. Nat. Plants 4, 245–246. 10.1038/s41477-018-0148-3 29725098

[B74] Weber A. P. M. Schwacke R. Flügge U.-I. (2005). Solute transporters of the plastid envelope membrane. Annu. Rev. Plant Biol. 56, 133–164. 10.1146/annurev.arplant.56.032604.144228 15862092

[B75] Wedel N. Soll J. (1998). Evolutionary conserved light regulation of Calvin cycle activity by NADPH-mediated reversible phosphoribulokinase/CP12/glyceraldehyde-3-phosphate dehydrogenase complex dissociation. Proc. Natl. Acad. Sci. USA. 95, 9699–9704. 10.1073/pnas.95.16.9699 9689144 PMC21402

[B76] Wenderoth I. Scheibe R. von Schaewen A. (1997). Identification of the cysteine residues involved in redox modification of plant plastidic glucose-6-phosphate dehydrogenase. J. Biol. Chem. 272, 26985–26990. 10.1074/jbc.272.43.26985 9341136

[B77] Wendt U. K. Hauschild R. Lange C. Pietersma M. Wenderoth I. von Schaewen A.. (1999). Evidence for functional convergence of redox regulation in G6PDH isoforms from cyanobacteria and higher plants. Plant Mol. Biol. 40, 487–494. 10.1023/A:1006257230779 10437832

[B78] Wendt U. K. Wenderoth I. Tegeler A. von Schaewen A. (2000). Molecular characterization of a novel glucose-6-phosphate dehydrogenase from potato (*Solanum tuberosum* L.). Plant J. 23, 723–733. 10.1046/j.1365-313x.2000.00840.x 10998184

[B79] Winkel B. S. (2004). Metabolic channeling in plants. Annu. Rev. Plant Biol. 55, 85–107. 10.1146/annurev.arplant.55.031903.141714 15725058

[B80] Xiong Y. DeFraia C. Williams D. Zhang X. Mou Z. (2009). Characterization of *Arabidopsis* 6-phosphogluconolactonase T-DNA insertion mutants reveals an essential role for the oxidative section of the plastidic pentose phosphate pathway in plant growth and development. Plant Cell Physiol. 50, 1277–1291. 10.1093/pcp/pcp070 19457984

[B81] Zacharias D. A. Violin J. D. Newton A. C. Tsien R. Y. (2002). Partitioning of lipid-modified monomeric GFPs into membrane microdomains of live cells. Science 296, 913–916. 10.1126/science.1068539 11988576

