# Supplementary material for: Alternative splicing of Arabidopsis G6PD5 recruits NADPH-producing OPPP reactions to the endoplasmic reticulum
Source: Front Plant Sci. 2022 Sep 2;13:909624. doi: 10.3389/fpls.2022.909624 (PMC9478949; doi:10.3389/fpls.2022.909624)
Supplement: Supplementary file 1 [file Table_1.DOCX]

**Supplemental Table S1.** Oligonucleotide primers used in this study.

| **No.** | **Name** | **Sequence** | **Construct/**  **Application** |
| --- | --- | --- | --- |
| ***G6PD5.1-5* (At3g27300)** | | | |
| 1443 | G6PD5 -Stopp as Acc65I | NNNGGTACCCAATGTAGGAGGGATCC | G6PD5.x-GFP/OFP  G6PD5.x-*ro*GFP  G6PD5.x-YFP^N/C^ |
| 1444 | G6PD5.4 s XbaI | NNNTCTAGAATGGTTTCACGTAATTATAATATTAG | G6PD5.4-GFP/OFP  GFP/OFP-G6PD5.4  G6PD5.4-*ro*GFP |
| 1445 | G6PD5.5 s XbaI | NNNTCTAGAATGAGACTCGAATTGTTGGAAAATG | G6PD5-roGFP/OFP |
| 1463 | N-term G6PD5 as | NNNggtaccCTTCCAATAGTTTTTTAACAC | N-term. 5.4/5.5-GFP/OFP |
| 1507 | G6PD5 as BglII | NNNAGATCTTTACAATGTAGGAGGG | GFP/OFP-G6PD5.1  GFP/OFP-G6PD5.4 |
| 1572 | G6PD5 8aa NdeI as | NNNCATATGCCATTGACCAGAACCCATC | G6PD5.5-GFP/OFP |
| 1593 | mRNA 5.4 s | CAATAATTCCCTTAATGGTTTCACGTAATT | RT-PCR G6PD5.4 |
| 1594 | mRNA 5.1/5.4 exon-span as | CAAAATACAGAACAAACCTGCGACGG | RT-PCR G6PD5.1/G6PD5.4 |
| 1595 | mRNA 5.5 exon-span s | CACCGTCGCAGTTATGTTATAGCCTAAG | RT-PCR G6PD5.5 |
| 1596 | mRNA 5.5 unique as | CAGAACAAACCTGTAAAATTGAAGTTTAGTG | RT-PCR G6PD5.5 |
| 1597 | mRNA 5.1 s | GTTTCACTACTATCCATTTACAAACAGTCCC | RT-PCR G6PD5.1 |
| ***PGL2* (At3g49360)** | | | |
| 549 | PGL2 SpeI s | NNNACTAGTNATGGCACCGGTGAAGAGGAG | GFP-PGL2, YFP^N/C^-PGL2 |
| 550 | PGL2 BamHI as | NNNGGATCCTCAAAGGATGGAGCACCAGC | GFP-PGL2 |
| 1140 | PGL2 C256S s | CTCCCTCATGGCTGGAGCTCCATCCTTTGAGG | GFP-PGL2 (C256S) |
| 1141 | PGL2 C256S as | CCTCAAAGGATGGAGCTCCAGCCATGAGGGAG | GFP-PGL2 (C256S) |
| ***PGD2* (At3g02360)** | | | |
| 714 | PGD2 SpeI s | NNNactagtATGGCTGTTCAACCTACAAG | GFP-PGD2 |
| 1342 | PGD2 BamHI as | NNNggatccTCAGATCTTAGATTGTCTTG | GFP-PGD2 |
| ***ATG8e* (At2g45170)** | | | |
| 1577 | ATG8e XbaI s | NNNtctagaATGAATAAAGGAAGCATCTTTAAG | GFP/OFP-ATG8e |
| 1578 | ATG8e BamHI as | NNNggatccTTAGATTGAAGAAGCACCG | GFP/OFP-ATG8e |
| ***C4H* ( At2g30490)** | | | |
| 1608 | C4H XbaI s | NNNtctagaATGGACCTCCTCTTGCTG | C4H-*ro*GFP |
| 1609 | C4H Acc65i as | NNNggtaccACAGTTCCTTGGTTTCATAAC | C4H-*ro*GFP |
| ***KCR1* (At1g67730)** | | | |
| 1610 | KCR1 XbaI s | NNNtctagaATGGAGATCTGCACTTACTTC | KCR1-*ro*GFP |
| 1611 | KCR1 Acc65i as | NNNggtaccTTCTTTCTTCATGGAGTC | KCR1-*ro*GFP |
| ***ATR1* ( At4g24520)** | | | |
| 1623 | ATR1 XbaI s | NNNtctagaATGACTTCTGCTTTGTATG | ATR1-*ro*GFP |
| 1624 | ATR1 Acc65i as | NNNggtaccCCAGACATCTCTGAG | ATR1-*ro*GFP |
| ***HXK2.1 (At2g19860.1)*** | | | |
| 1720 | HXK2.1 XhoI s | NNNctcgagATGGGTAAAGTGGCAGTTGC | HXK2.1-*ro*GFP |
| 1721 | HXK2 -Stopp Acc65I as | NNNggtaccACTTGTTTCAGAGTCATCTTC | HXK2.1-*ro*GFP |
| ***Housekeeping Genes*** | | | |
| 1562 | EF-1α s | ACCAAGTACTACTGCACAGTCATTG | RT-PCR |
| 1563 | EF-1α as | CTTCAAAAACTTGGGCTCCTTCT | RT-PCR |
| 1733 | UBQ10 s | GATCTTTGCCGGAAAACAATTGGAGGATGGT | RT-PCR |
| 1734 | UBQ10 as | CGACTTGTCATTAGAAAGAAAGAGATAACAG | RT-PCR |
| **Vectors** | | | |
| 1009 | GFP A206K s | CTGTCCACACAATCTaagCTTTCGAAAGATCCC | monomeric GFP |
| 1010 | GFP A206K as | GGGATCTTTCGAAAGcttAGATTGTGTGGACAG | monomeric GFP |
| 1103 | GFP +NotI s | Ccaagcggccgcgccaagc | Introduction of a NotI site |
| 1104 | GFP +NotI as | gcttggcgcggccgcttgG |  |
| 1249 | OFP ΔPstI s | CAGGACTCCTCCCTaCAGGACGGCGAGTTC | Removal of an internal PstI site |
| 1250 | OFP ΔPstI as | GAACTCGCCGTCCTGtAGGGAGGAGTCCTG |  |
| 1202 | 35S Prom NotI s | NNNgcggccgcTGAGACTTTTCAACAAAGG | Cloning of double cassette constructs |
| 1203 | NosT SbfI as | NNNcctgcaggCCGATCTAGTAACATAGATGAC |  |

Abbreviations: s = sense; as = antisense
