# Supplementary material for: Alternative splicing of Arabidopsis G6PD5 recruits NADPH-producing OPPP reactions to the endoplasmic reticulum
Source: Front Plant Sci. 2022 Sep 2;13:909624. doi: 10.3389/fpls.2022.909624 (PMC9478949; doi:10.3389/fpls.2022.909624)
Supplement: Supplementary file 3 [file Data_Sheet_1.pdf]

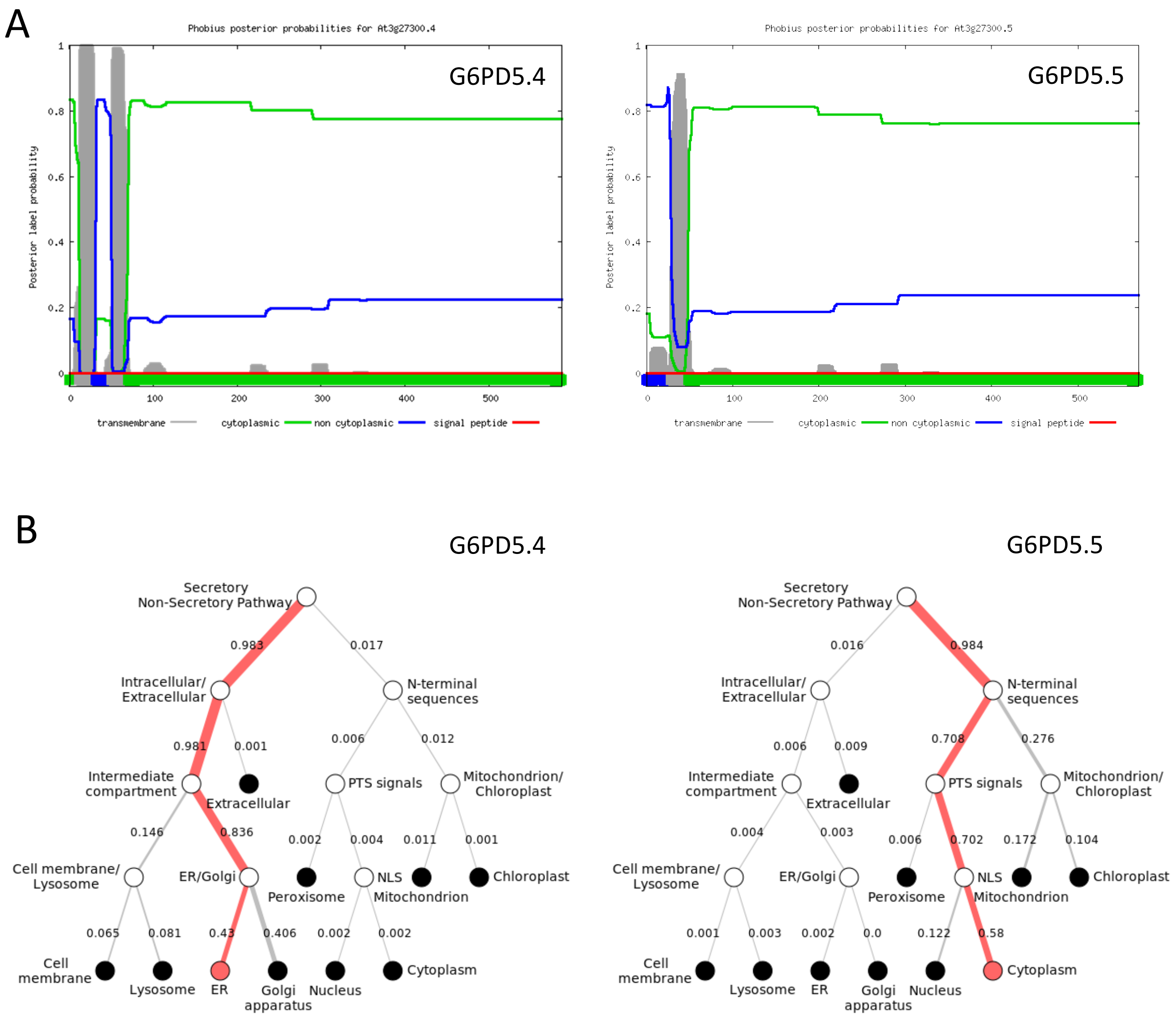

Suppl. Figure S1. Predicted topology and localization for alternative proteoforms G6PD5.4 and G6PD5.5. Bioinformatic analyses supporting Figure 2. **A**, Hydropathy plots (of Phobius) predict two membrane-spanning domains (grey) for G6PD5.4 (with both N- and C-terminus in the cytosol, green) and one for G6PD5.5, listing the N-terminus as non-cytoplasmic (blue), but without signal peptide (red, see legend). **B**, Predicted subcellular localization (of DeepLoc-1.0) for G6PD5.4 is the endoplasmic reticulum (ER, left) and for G6PD5.5 the cytosol (Cytoplasm, right).

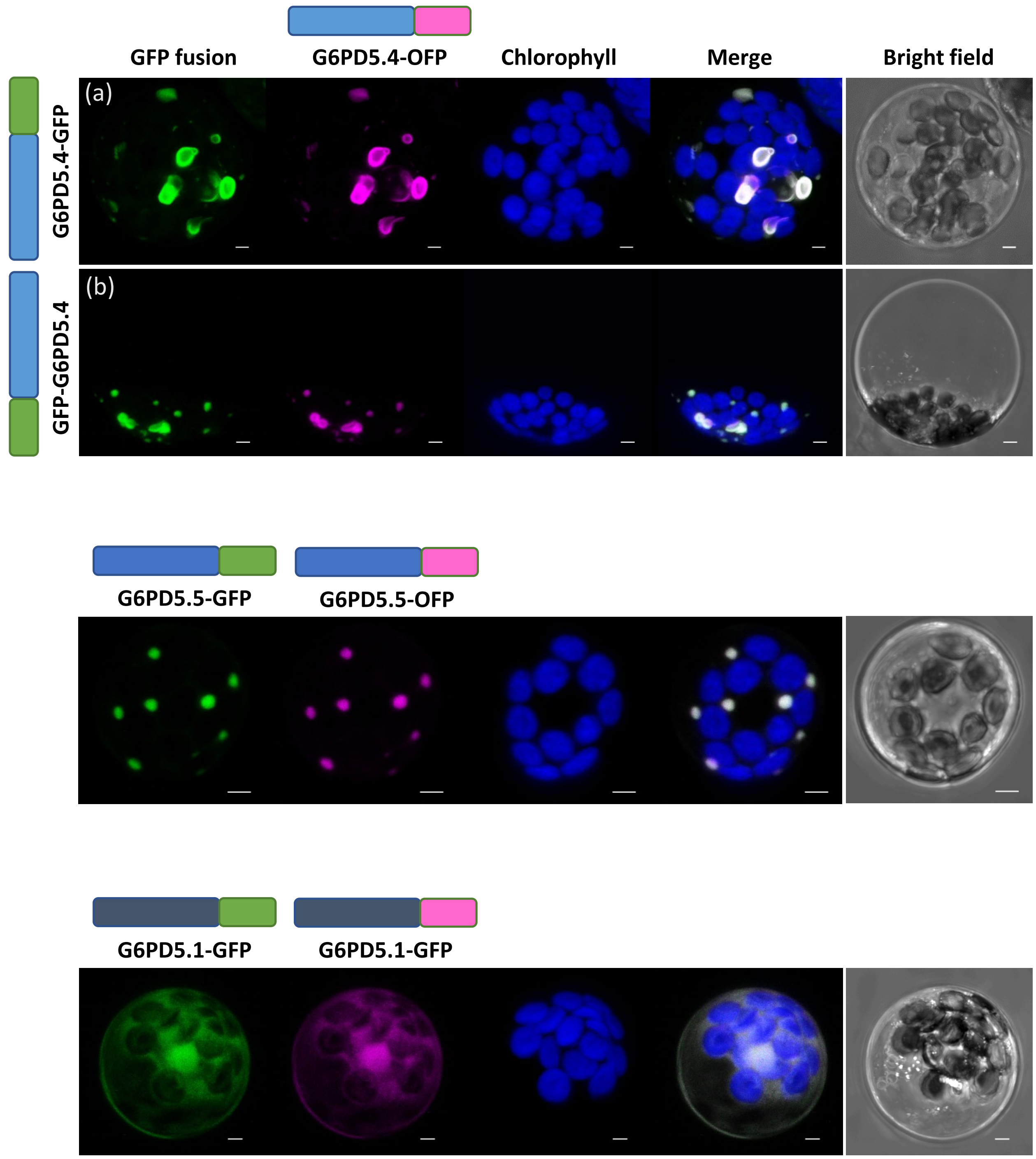

Suppl. Figure S2. Co-expression of the <u>same</u> *G6PD5* splice variants as GFP- and OFP-reporter fusions. Single channel images of Figure 3B. Top, co-expression of the G6PD5.4-reporter combinations (N-in); center, co-expression of the only possible G6PD5.5 combination (N-out); bottom, co-expression of G6PD5.1-reporter fusions. The images show maximal projections of about 30 optical single sections. GFP in green, OFP in magenta, and chlorophyll autofluorescence in blue; white signals, co-localization of GFP and OFP (or very close signals <200 nm). Scale bars 3 µm.

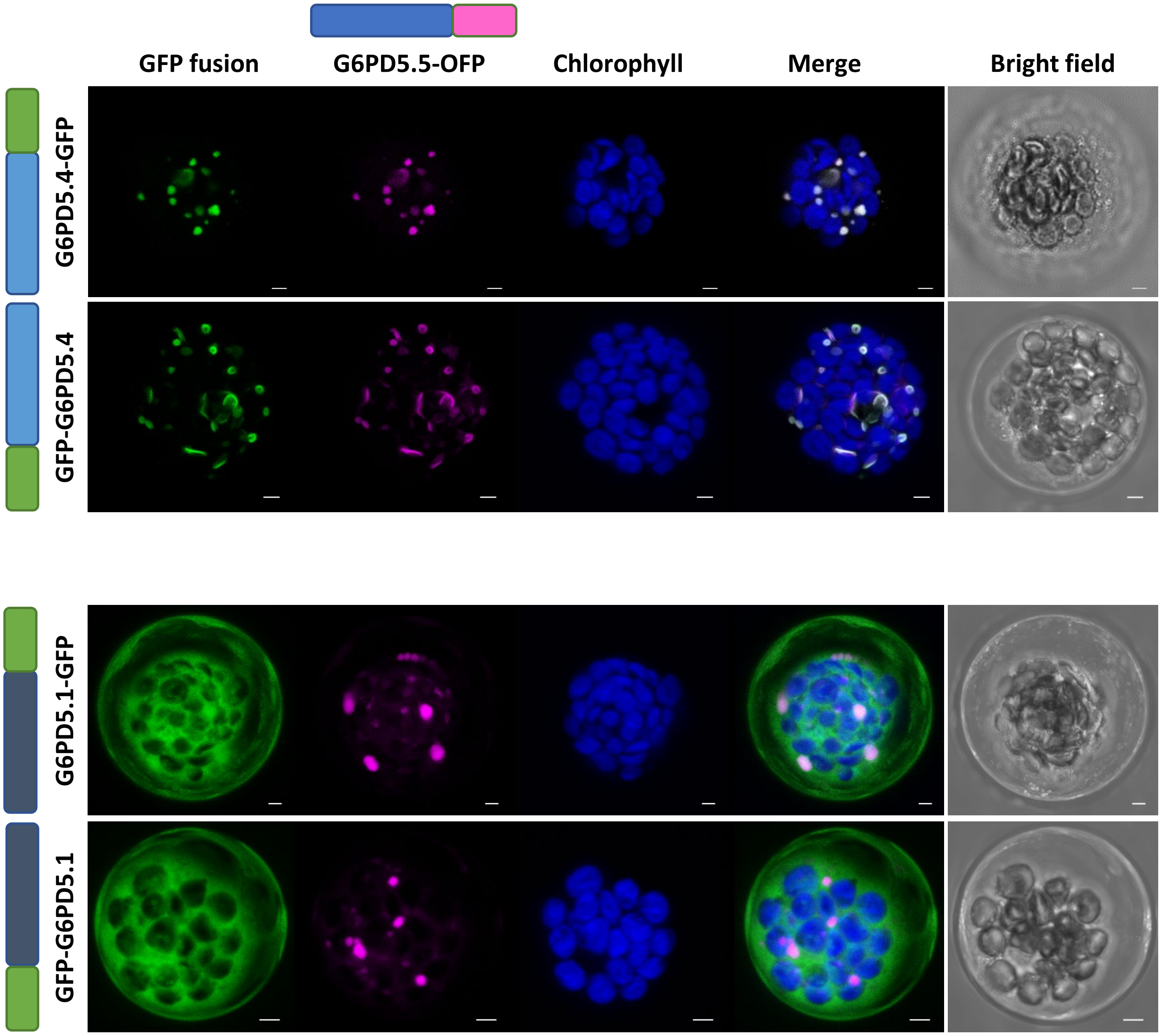

Suppl. Figure S2. (continued) Co-expression of <u>different</u> G6PD5 splice variants as GFP- and OFP-reporter fusions. Single channel images of Figure 3C. Co-expression of G6PD5.5-OFP with the GFP fusions of G6PD5.4 (top) or G6PD5.1 (bottom). The images show maximal projections of about 30 optical single sections. GFP in green, OFP in magenta, and chlorophyll autofluorescence in blue; white signals, co-localization of GFP and OFP (or very close signals <200 nm). Scale bars 3 µm.

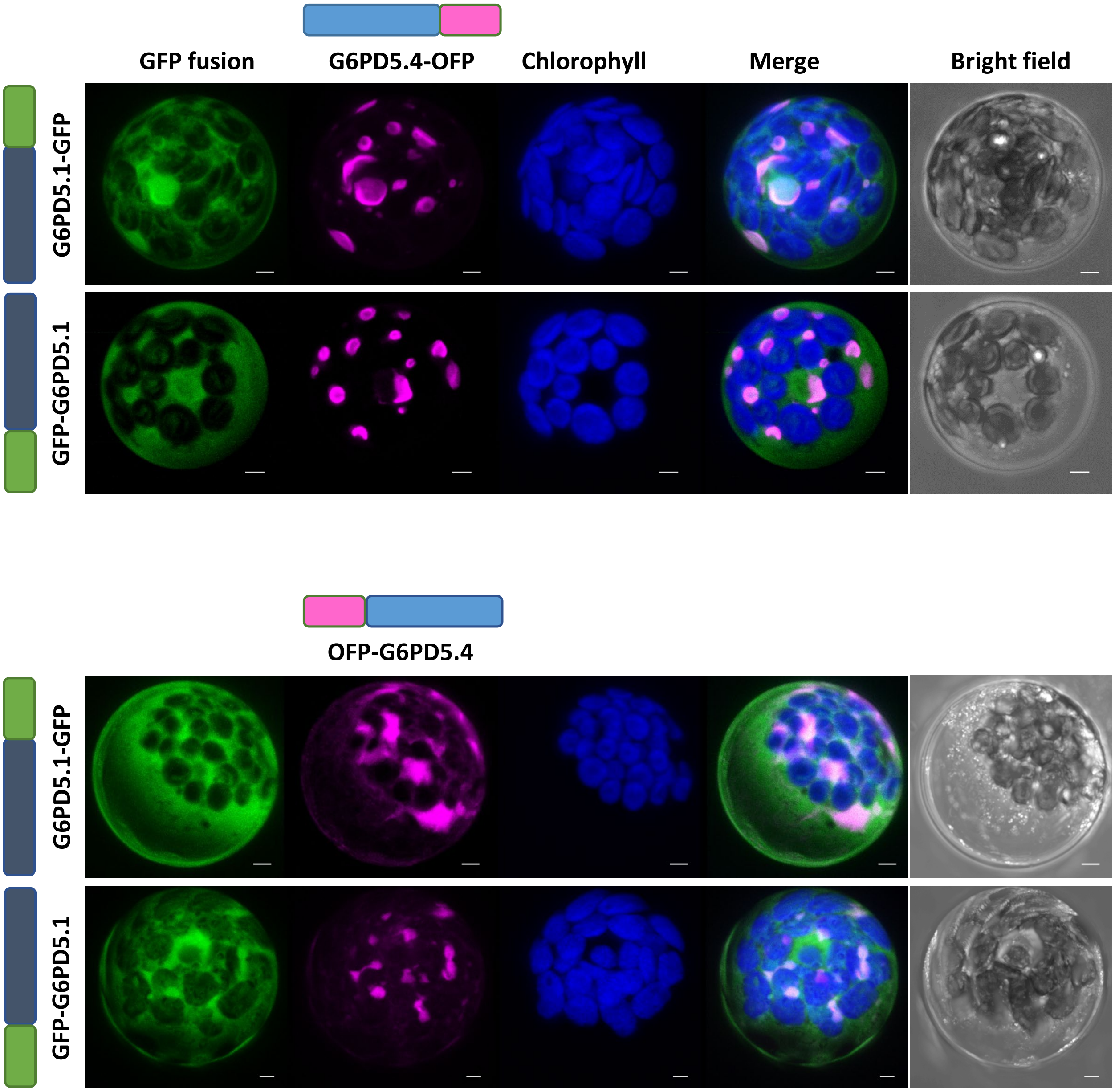

Suppl. Figure S2. (continued) Co-expression of <u>different</u> G6PD5 splice variants as GFP- and OFP-reporter fusions. Single channel images of Figure 3D. Co-expression of G6PD5.4-OFP or OFP-G6PD5.4 with the GFP fusions of G6PD5.1. The images show maximal projections of about 30 optical single sections. GFP in green, OFP in magenta, and chlorophyll autofluorescence in blue; white signals, co-localization of GFP and OFP (or very close signals <200 nm). Scale bars 3 µm.

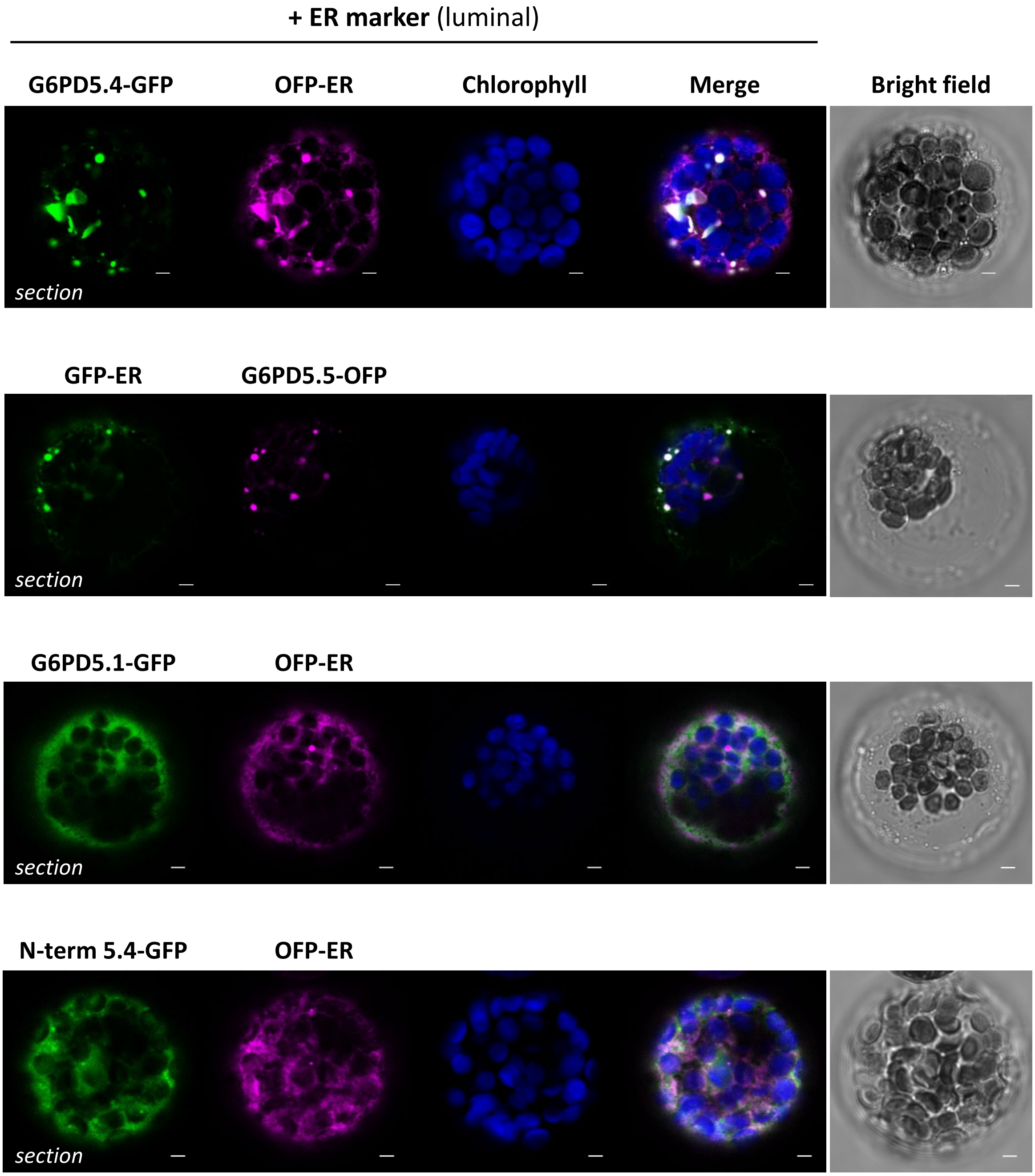

Suppl. Figure S3. Subcellular localization analysis of membrane-bound G6PD5.4 and G6PD5.5.
Single channel images of Figure 4A. Co-expression of the indicated G6PD5-reporter fusions with a luminal ER marker of opposite colour (GFP-ER or OFP-ER). The images show single optical sections. GFP in green, OFP in magenta, and chlorophyll autofluorescence in blue; white signals, co-localization of GFP and OFP (or very close signals <200 nm). Scale bars, 3 µm.

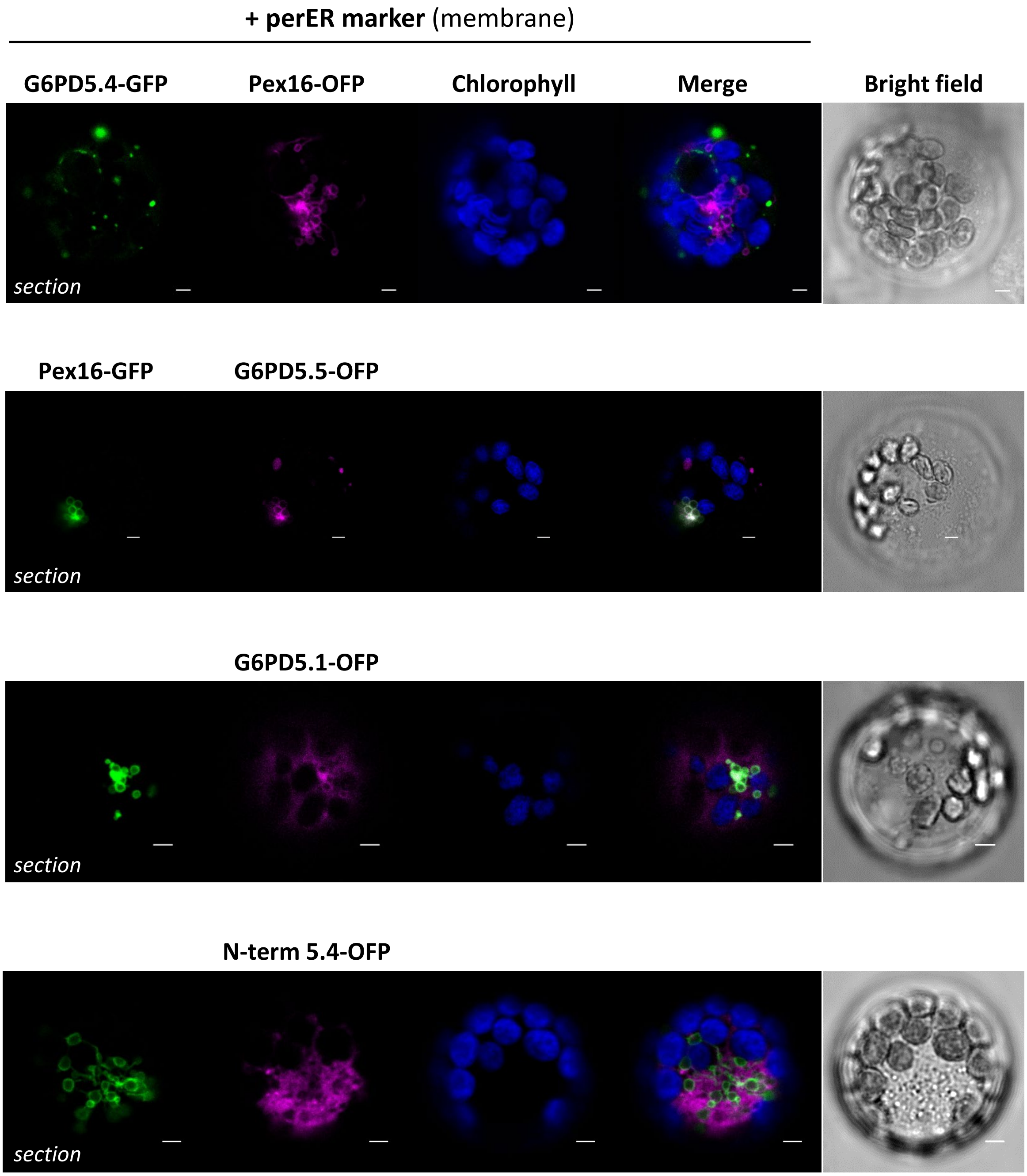

Suppl. Figure S3 (continued). Subcellular localization analyses of membrane-bound G6PD5.4 and G6PD5.5. Single channel images of Figure 4B. Co-expression of the indicated G6PD5-reporter fusions with a membrane-bound peroxisomal ER marker of opposite colour (Pex16-GFP or Pex16-OFP). The images show single optical sections. GFP in green, OFP in magenta, and chlorophyll autofluorescence in blue; white signals, co-localization of GFP and OFP (or very close signals <200 nm). Scale bars, 3 µm.

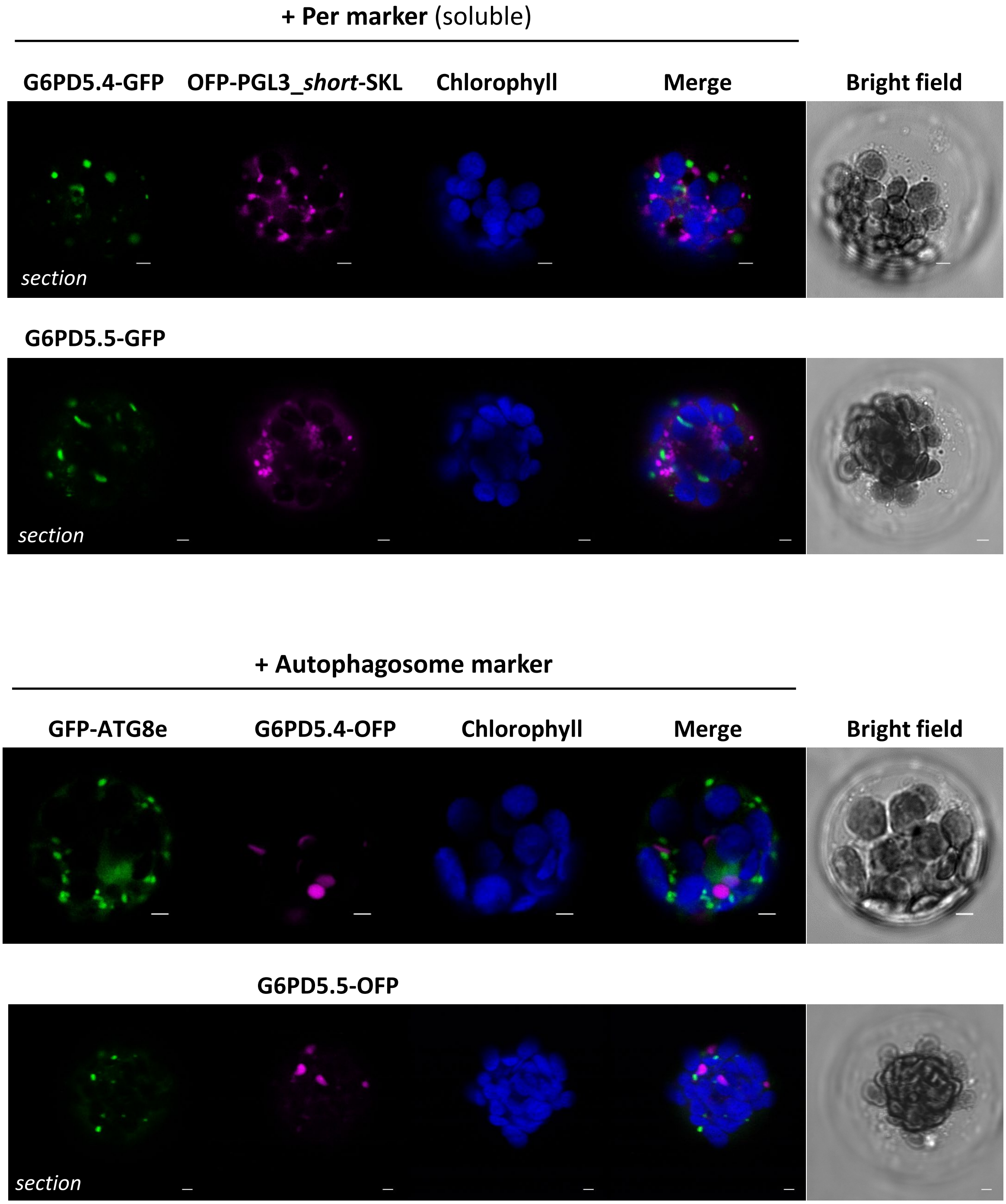

Suppl. Figure S3 (continued). Subcellular localization analyses of membrane-bound G6PD5.4 and G6PD5.5. Single channel images of Figure 4C. Co-expression of the indicated G6PD5-reporter fusions with a soluble peroxisome marker of opposite colour (GFP- or OFP-PGL3_*short*-SKL), or an autophagosome marker of opposite colour (GFP- or OFP-ATG8e). The images show 3D-projections of about 30 optical sections. GFP in green, OFP in magenta, and chlorophyll auto-fluorescence in blue; white signals, co-localization of GFP and OFP (or very close signals <200 nm). Scale bars, 3 µm.

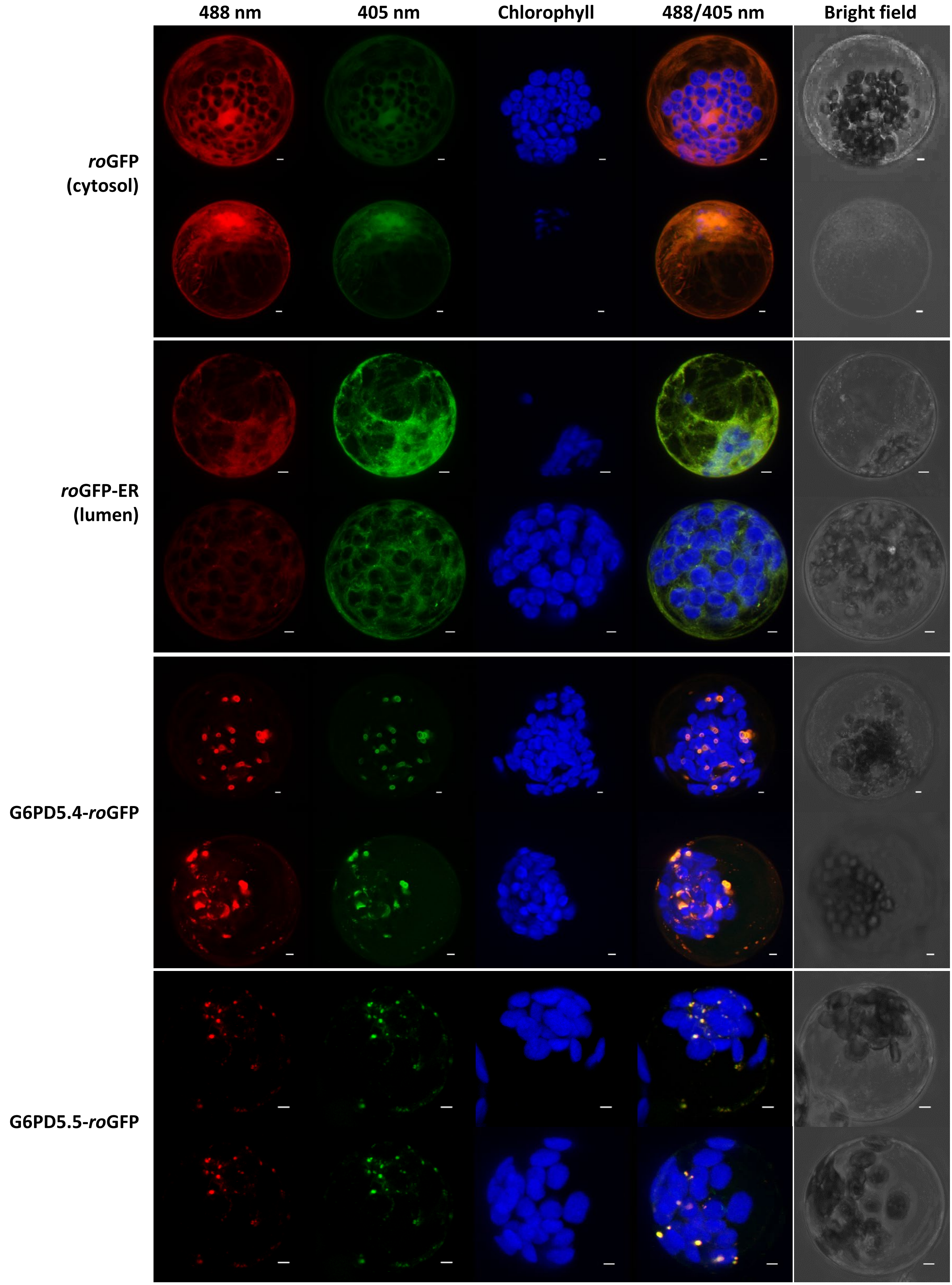

Suppl. Figure S4. Topology analyses of membrane-bound G6PD5.4 and G6PD5.5 with C-terminally fused *ro*GFP. Single channel images of Figure 5A. Two representative cells are shown for each construct. The 488/405 ratios resemble the cytosolic *ro*GFP (red-orange/yellow) and not luminal *ro*GFP-ER (green). Scale bars, 3 µm.

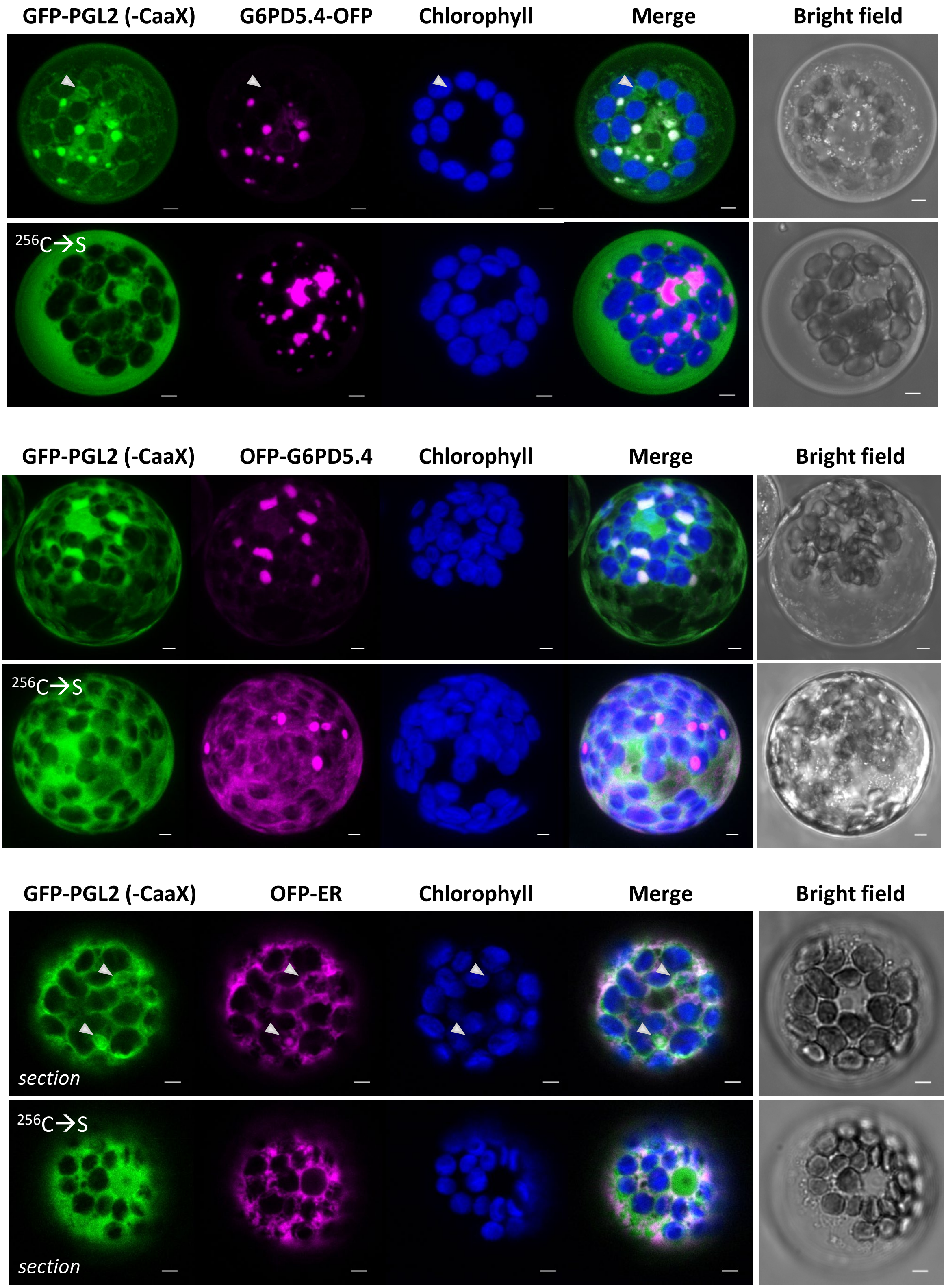

Suppl. Figure S5. Co-expression of G6PD5.4-OFP with GFP-PGL2 or PGL2 with mutated -CaaX motif (-SSIL). Single channel images of Figure 6B. White arrowheads point to regions with extra membrane material labeled by PGL2. Note that the C-to-S mutation in the C-terminal CaaX motif renders PGL2 entirely cytosolic. The images show 3D-projections of about 30 optical sections. GFP in green, OFP in magenta, and chlorophyll autofluorescence in blue; white signals, co-localization of GFP and OFP (or very close signals <200 nm). Scale bars, 3 µm.

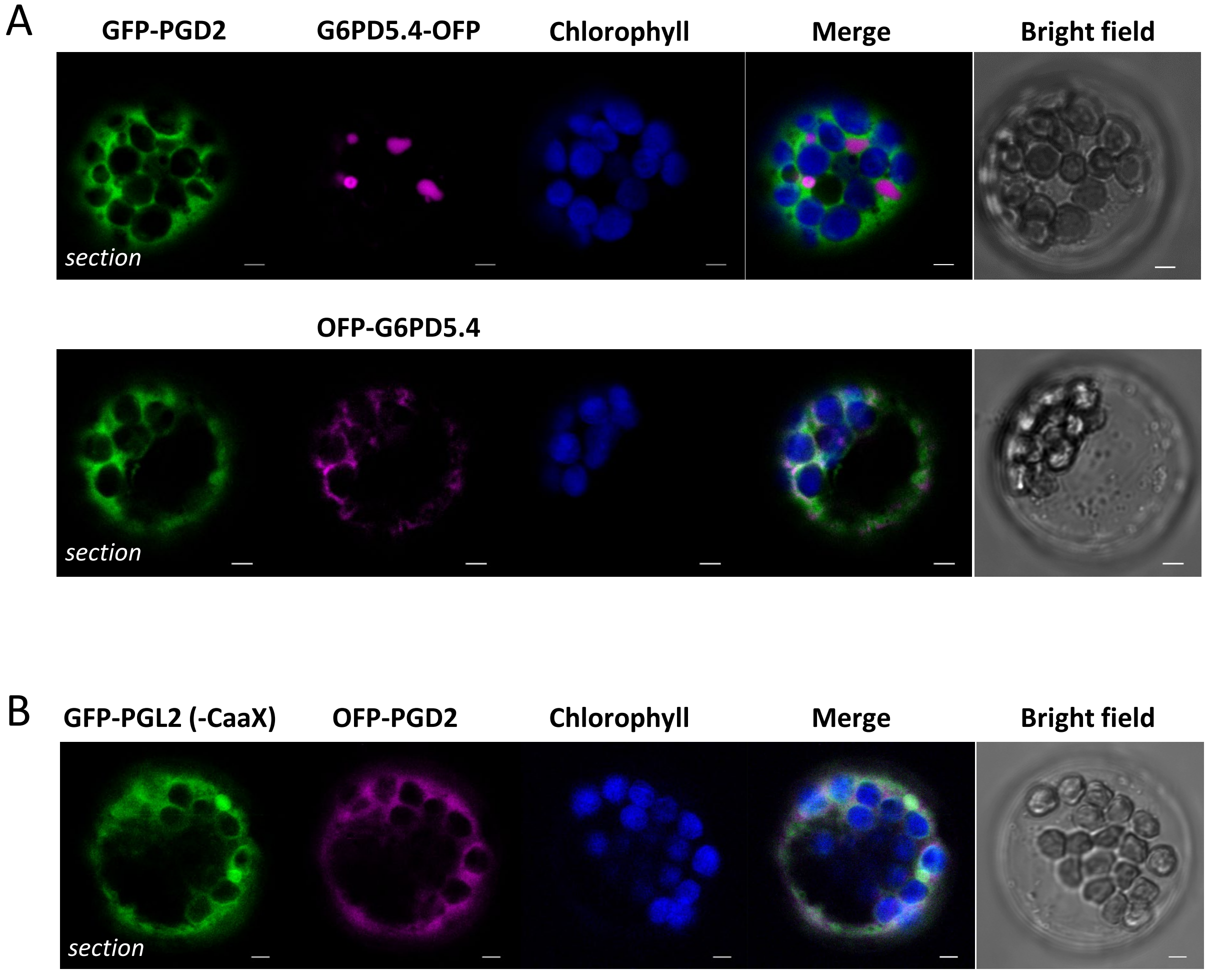

Suppl. Figure S6. Co-expression of the G6PD5.4 or PGL2 fusions with N-terminally tagged PGD2.
6-phosphogluconate dehydrogenase (PGD) is the other OPPP dehydrogenase contributing to NADPH formation. All Arabidopsis PGD isoforms were co-expressed as GFP-reporter fusions, but none showed clear co-localization with G6PD5.4-OFP, here shown for PGD2 (with additional palmitoylation site; Figure 6A). **A**, GFP-PGD2 co-expressed with G6PD5.4-OFP (top, no overlap), and with OFP-G6PD5.4 (bottom, some overlap). **B**, PGD2 was also tested with PGL2: membrane-bound GFP-PGL2 (-CaaX) co-expressed with OFP-PGD2, showing weak overlap (likely at the ER). The images show single optical sections. GFP in green, OFP in magenta, and chlorophyll autofluorescence in blue; white signals, co-localization of GFP and OFP (or very close signals <200 nm). Scale bars, 3 µm.

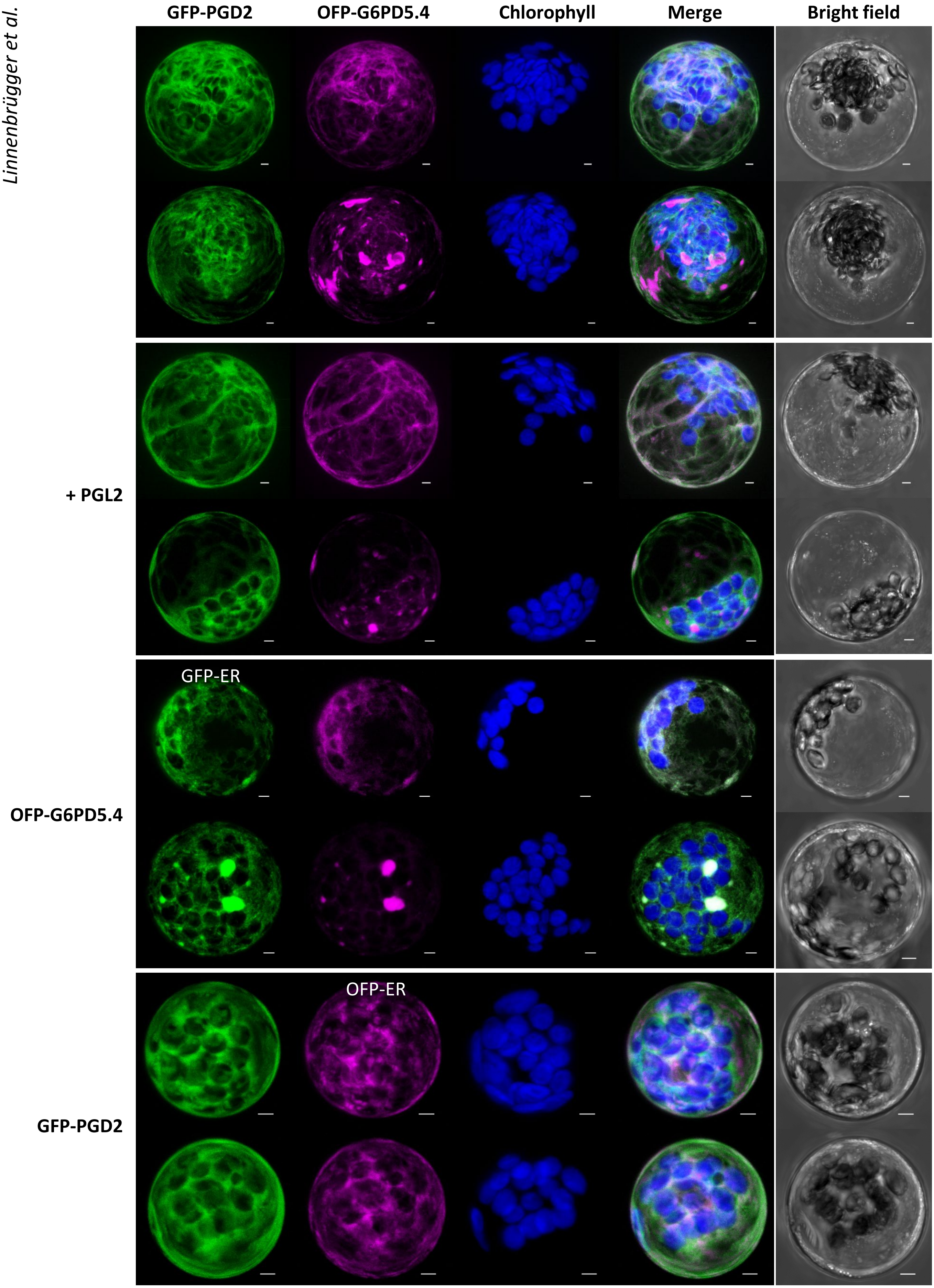

Suppl. Figure S7. Single channel images of Figure 7.
Co-expression of G6PD5.4 with PGD2 in the absence or presence of PGL2 in Arabidopsis wildtype protoplasts. OFP-G6PD5.4 and GFP-PGD2 were also co-expressed with the luminal ER marker (GFP- or OFP-ER, bottom). The images show maximal projections of about 30 optical sections. GFP in green, OFP in magenta, and chlorophyll autofluorescence in blue; white signals indicate co-localization of GFP and OFP (or very close signals <200 nm). Scale bars, 3 µm.

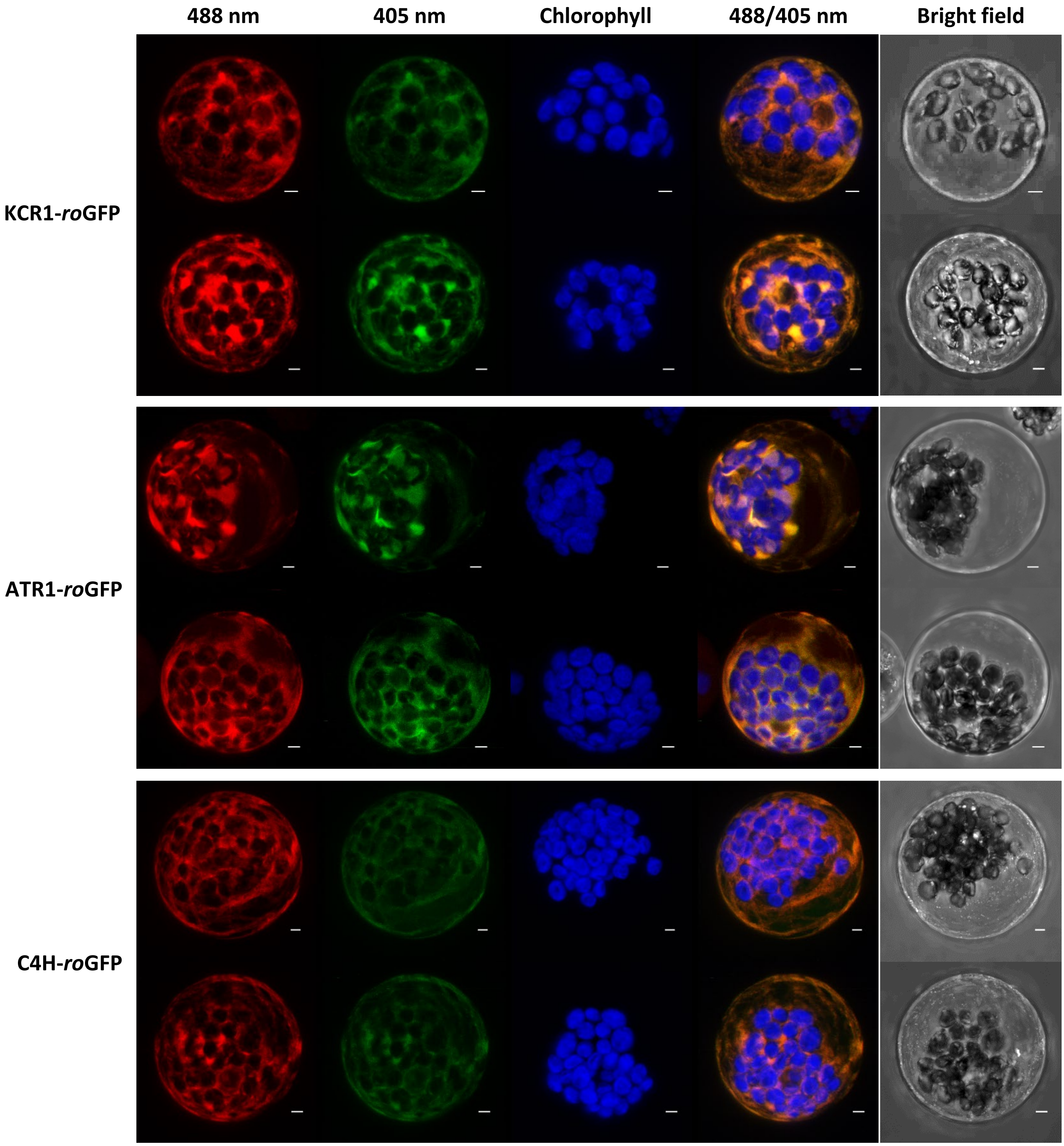

Suppl. Figure S8. Topology analyses of NADPH-dependent enzymes with C-terminally fused *ro*GFP at the ER. Experimental support for Figures 9. Note that the 488/405 ratios resemble cytosolic *ro*GFP (red-orange to yellow in Figure 5A), confirming that the catalytic domains of these ER-bound enzymes face the cytosol. Scale bars, 3 µm.

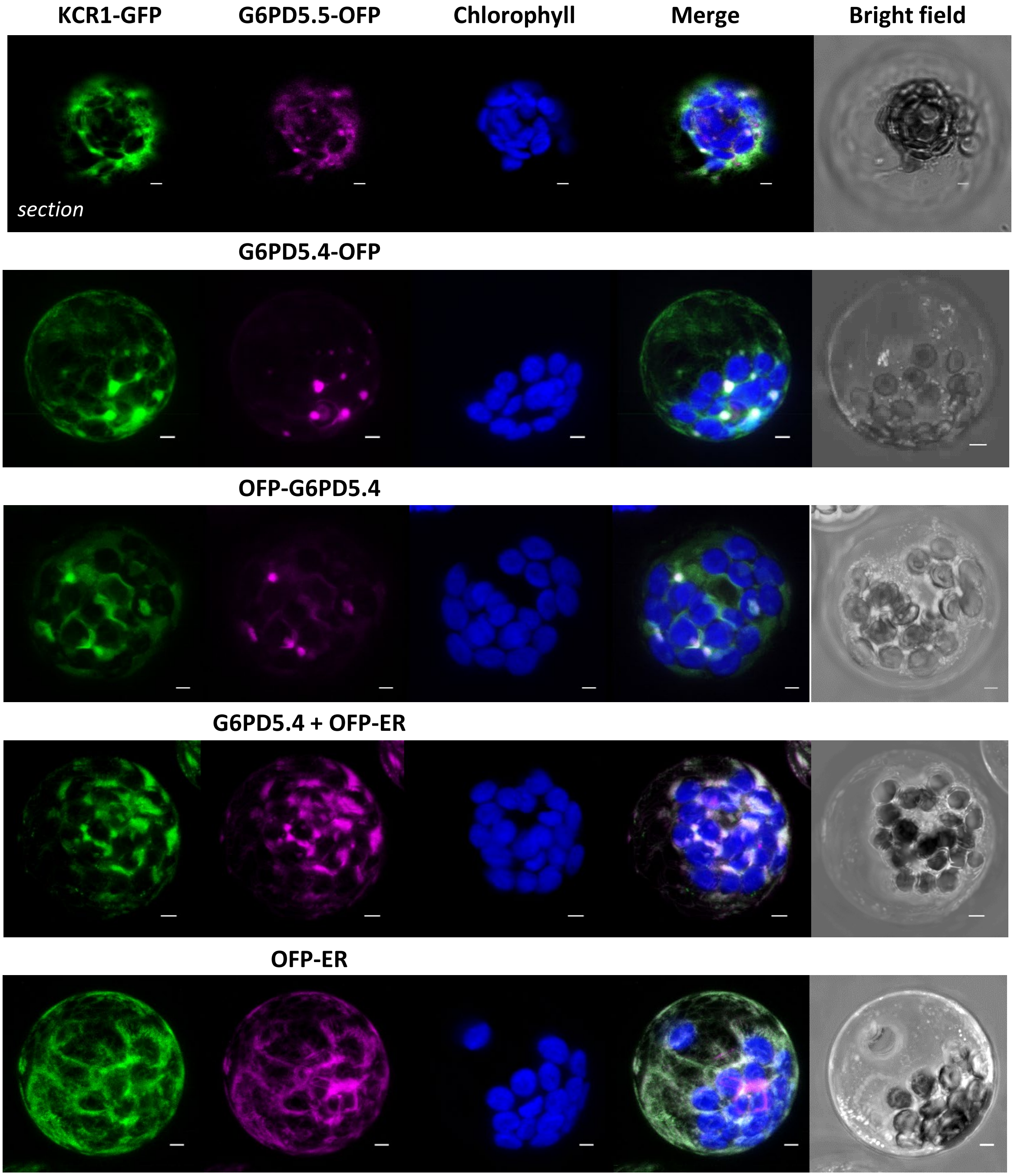

Suppl. Figure S9. Single channel images of Figure 9B.
KCR1-GFP (ketoacyl-CoA reductase, a component of the fatty acid elongase complex at the ER), was co-expressed with the indicated G6PD5 variants. If not indicated, the images show maximal projections of about 30 single optical sections. GFP in green, OFP in magenta, and chlorophyll autofluorescence in blue; white signals indicate co-localization of GFP and OFP (or very close signals <200 nm). Scale bars, 3 µm.

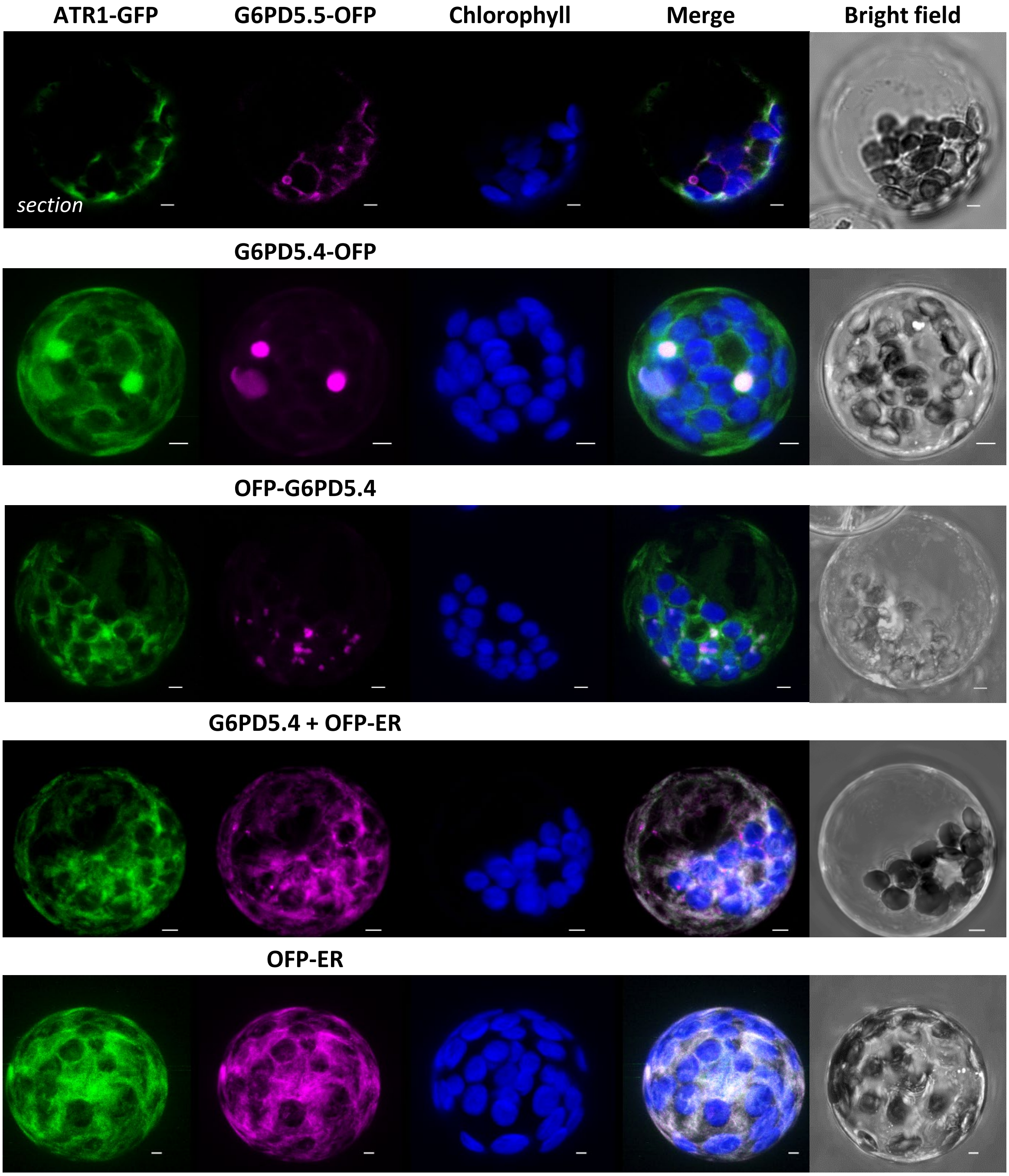

Suppl. Figure S9 (continued). Single channel images of Figure 9B.
ATR1-GFP (NADPH:cytochrome P450 oxidoreductase1 that reduces cytochrome P450 enzymes at the ER), was co-expressed with the indicated G6PD5 variants. If not indicated, the images show maximal projections of about 30 single optical sections. GFP in green, OFP in magenta, and chlorophyll autofluorescence in blue; white signals indicate co-localization of GFP and OFP (or very close signals <200 nm). Scale bars, 3 µm.

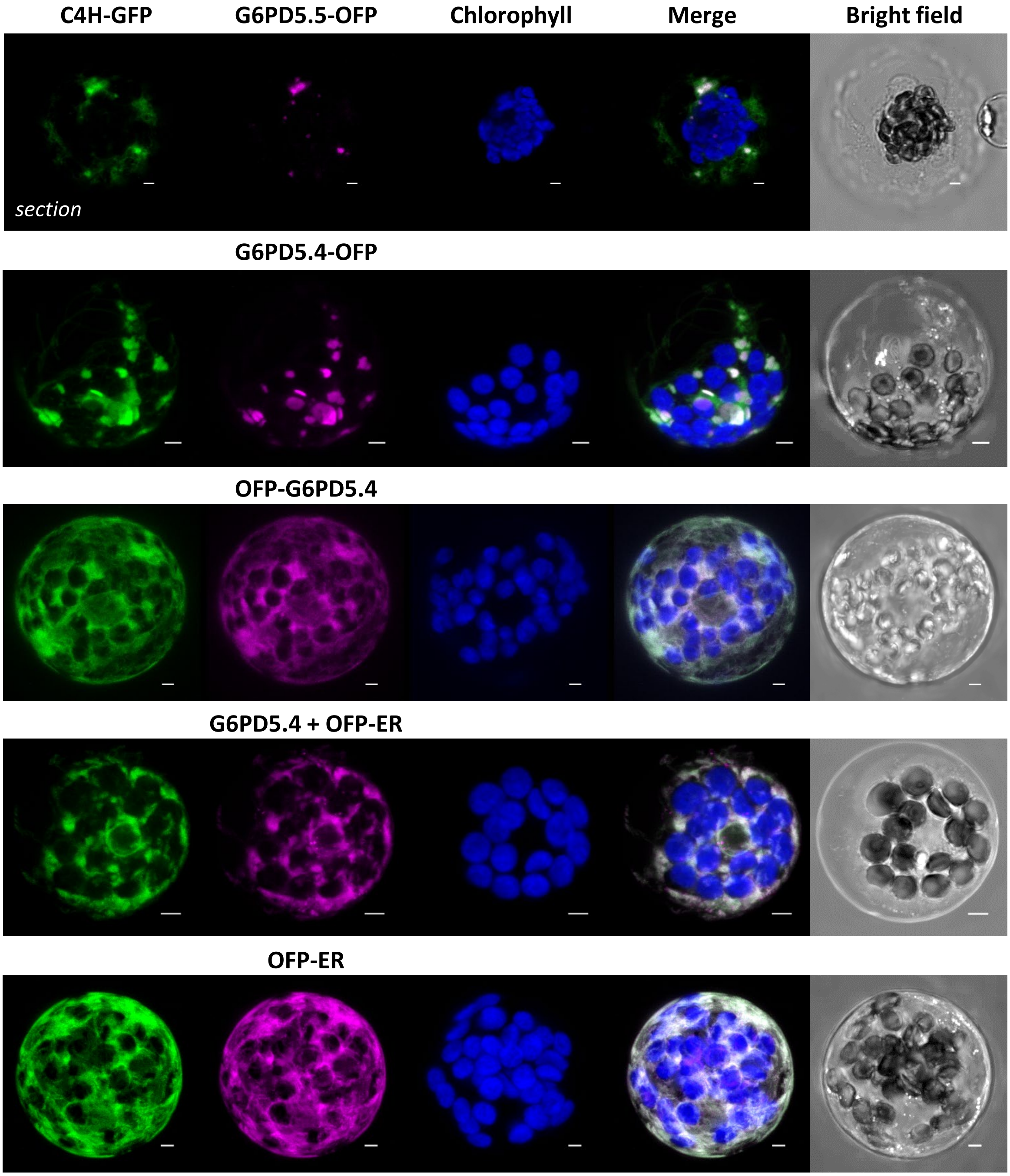

Suppl. Figure S9 (continued). Single channel images of Figure 9B.
C4H-GFP (cinnamate 4-hydroxylase/CYP73A5) involved in phenylpropane biosynthesis at the ER), was co-expressed with the indicated G6PD5 variants. If not indicated, the images show maximal projections of about 30 single optical sections. GFP in green, OFP in magenta, and chlorophyll autofluorescence in blue; white signals indicate co-localization of GFP and OFP (or very close signals <200 nm). Scale bars, 3 µm.

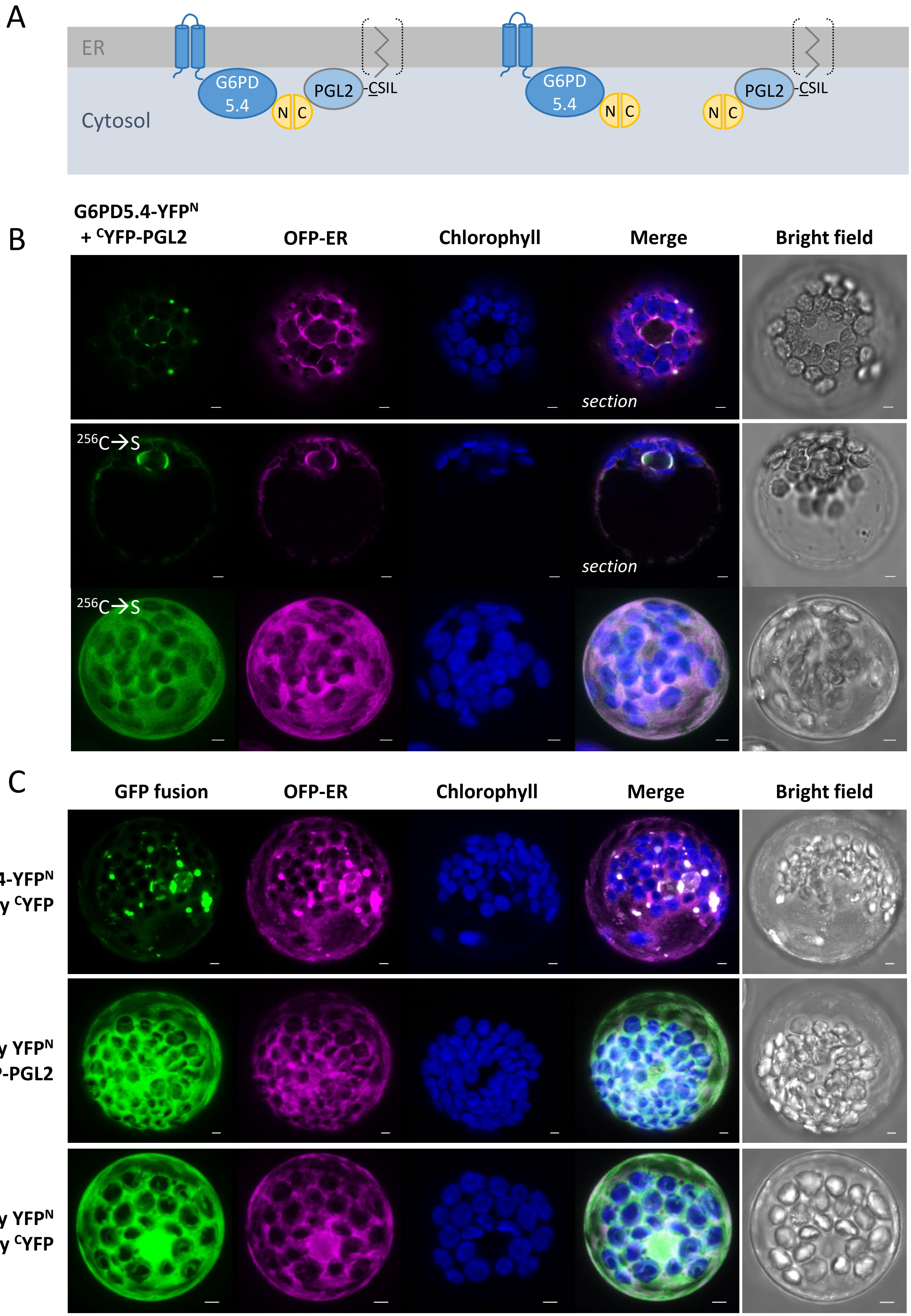

Suppl. Figure S10. BiFC analyses of split YFP-fusion constructs G6PD5.4-YFP^{N} and ^{C}YFP-PGL2.
A, Scheme of split YFP reconstitution between G6PD5.4-YFP^{N} and ^{C}YFP-PGL2 with C-terminal CaaX motif or without (^{256}C→S mutation, bracket). **B**, G6PD5.4-YFP^{N} and ^{C}YFP-PGL2 were co-expressed with a soluble ER marker (OFP-ER). **C**, Co-expression of each construct with the corresponding empty vector (controls). Note that although using non-overlapping split YFP versions, also the empty vector combination (bottom) gave rise to YFP signals. Scale bars, 3 µm.

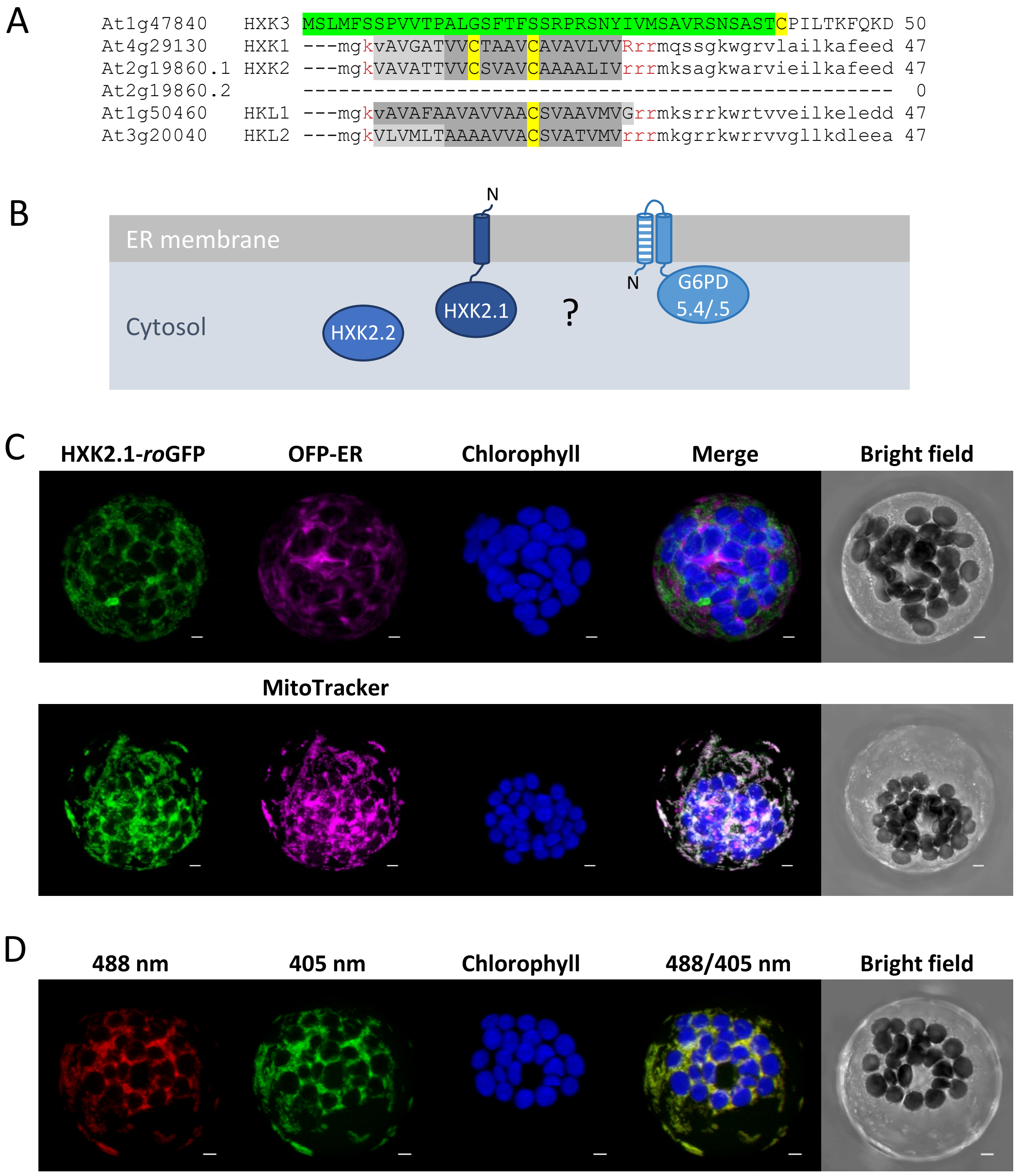

Suppl. Figure S11. Localization analyses of alternative splice form hexokinase 2.1.
A, N-terminal part of an alignment of highly active hexokinase (HXK) and hexokinase-like (HKL) isoforms – of which the latter lack glucokinase activity (Karve et al. 2008). There, HXK3-GFP localized at chloroplasts (transit peptide in green), HXK1-, HXK2-, and HKL2/HXK4-GFP fusions (membrane domains in grey) showed local accumulations, and HKL1-GFP overlapped with MitoTracker red. **B**, Scheme of soluble HXK2.2 and membrane-bound HXK2.1 that may provide G6P substrate to G6PD5.4 and G6PD5.5 at the ER (question mark). **C**, Co-expression of HXK2.1-*ro*GFP with the luminal ER marker (OFP-ER) in wild-type protoplasts showed no overlap, but HXK2.1-*ro*GFP expressing cells were stained by MitoTracker orange. The N-terminally shorter HXK2.2-*ro*GFP variant did not give any signal (not shown). The images show maximal projections of about 30 optical sections as merge of all channels. GFP in green, OFP or MitoTracker in magenta, and chlorophyll auto-fluorescence in blue. White signals indicate co-localization of GFP and OFP/MitoTracker (or very close signals <200 nm). **D**, Ratiometric analyses of HXK2.1-*ro*GFP hint at presence in an oxidizing environment (compare to the cytosolic and ER luminal controls in Suppl. Figure S4). Scale bars, 3 µm.
